# Cognition from the Body-Brain Partnership: Exaptation of Memory

**DOI:** 10.1146/annurev-neuro-101222-110632

**Published:** 2023-03-14

**Authors:** György Buzsáki, David Tingley

**Affiliations:** 1 Neuroscience Institute and Department of Neurology, NYU Grossman School of Medicine, New York University, New York, NY, USA;; 2 Center for Neural Science, New York University, New York, NY, USA; 3 Division of Endocrinology, Diabetes and Metabolism, Beth Israel Deaconess Medical Center, Harvard Medical School, Boston, Massachusetts, USA

**Keywords:** hippocampus, memory, sharp-wave ripples, homeostasis, allostasis, body control, glucose regulation

## Abstract

Examination of cognition has historically been approached from language and introspection. However, human language–dependent definitions ignore the evolutionary roots of brain mechanisms and constrain their study in experimental animals. We promote an alternative view, namely that cognition, including memory, can be explained by exaptation and expansion of the circuits and algorithms serving bodily functions. Regulation and protection of metabolic and energetic processes require time-evolving brain computations enabling the organism to prepare for altered future states. Exaptation of such circuits was likely exploited for exploration of the organism’s niche. We illustrate that exploration gives rise to a cognitive map, and in turn, environment-disengaged computation allows for mental travel into the past (memory) and the future (planning). Such brain-body interactions not only occur during waking but also persist during sleep. These exaptation steps are illustrated by the dual, endocrine-homeostatic and memory, contributions of the hippocampal system, particularly during hippocampal sharp-wave ripples.

There are these two young fish swimming along, and they happen to meet an older fish swimming the other way, who nods at them and says, “Morning, boys. How’s the water?” And the two young fish swim on for a bit, and then eventually one of them looks over at the other and goes, “What the hell is water?”

—David Foster Wallace (Kenyon College, 2005)

## INTRODUCTION

The old fish’s question is relevant to neuroscience. As neuroscientists plunge deeper into understanding dynamics of the brain, we often take it for granted that the brain is nested in the body and that this embeddedness has directed the evolution of brain function since the earliest nervous systems. While it has been acknowledged in cognitive science that one must take into account the embeddedness of the brain in the body and its environmental niche to understand cognition [embodied mind (Brooks 1991, Clark 1997, Gibbs & Raymond 2005, Gibson 1966)], this important view has not effectively penetrated neuroscience research and has faded over time.

The dominant framework promoting the view that we can understand neuronal circuits by probing them with sensory stimuli diminished the urgency of carefully attending to the importance of brain-body relationships. The alleged primacy of the commanding outside stimuli is also reflected by the psychological definition of cognition, “all the processes by which the sensory input is transformed, reduced, elaborated, stored, recovered and used” (Neisser 1976, pp. 4–5), which is often claimed as a uniquely human endeavor and continues to influence current thinking (Passingham et al. 2002).

The body is the most private and constant niche of the brain, with which it continuously interacts in a seamless manner without knowing it. This nestedness is a neuroecological continuum (Hegarty & Waller 2004, Northoff 2018), apparent only to a third-party observer. Below, we review experiments that demonstrate that understanding this deep bidirectional relationship is essential for virtually all aspects of brain functions, from the most basic to the most complex. Additional comments and references are available in the Supplemental Appendix.

## MIND-BRAIN-BODY

Although nervous systems evolved in parallel with ever more complex organ systems through natural selection, paradoxically, neuroscience inherited the brain as the substrate of its study through humankind’s quest for the spirit, soul, and the conscious mind (Chalmers 1997). Early thinkers portrayed the mind as a dedicated organ for learning about the true nature of the world through our sensors, which allow us to perceive signals and correctly interpret them. Largely for this reason, the emerging field of neuroscience followed a road map that began its investigation from a human mind–centric point of view and attempted to merge mental phenomena onto the physical, resulting in thinking matter. “Perceptual decisions entail translating sensory information into judgments, beliefs, or actions” (O’Connell & Kelly 2021, p. 496). As a result, the introspection-rooted terms of perceive, think, decide, attend, and remember have become among the most prominent terms in contemporary neuroscience.

This outside-in framework, with its human language–dependent definitions, is largely responsible for the practice of investigating brain circuits under body-constrained situations (Buzsáki et al. 2022, Cisek & Hayden 2022). Interest in the analysis of behavior has declined (Gomez-Marin & Ghazanfar 2019), and head-fixed and eye-fixed preparations have become encouraged mainstream methods in today’s neuroscience (see Supplemental Figure 3) (Luo et al. 2008). As a consequence, mechanisms of how vision and perceptual systems in general operate in the natural world have remained unexplored (Parker et al. 2020). At the same time, the roles of sleep and other nonconscious brain functions have been ignored for mental operations.

Frameworks matter. They bias how we design our experiments and how we interpret what we find (Chang 2007). Everything we measure in the brain is related to something else and is done with human-conceived instruments with a socially agreed-upon scale. If the reference is cognition or the human mind, we may find correlated brain activity without knowing whether those activity patterns are actually used by some reader/interpreter mechanisms in the brain. The goal of our review is to highlight how the same brain patterns viewed from different reference points inevitably lead to different interpretations and that using multiple references is necessary to understand the utility and physiological meaning (i.e., impact) of those patterns.

## BODY-BRAIN-MIND

### Grounding: A Case for the Body

Work performed under the outside-in framework has limitations because the process of investigation requires the artificial insertion of a privileged human experimenter who can observe and compare both the events in the world and activity in the brain. However, neurons that respond to these events cannot intrinsically represent anything because they cannot relate or compare their firing to something else. In contrast to the observing human, neurons in, for example, sensory areas driven by environmental signals alone cannot ground their activity to anything meaningful. Grounding refers to the ability of the brain’s circuits to assign meaning to changes in neuronal firing patterns that result from sensory inputs.

**Figure 1:**
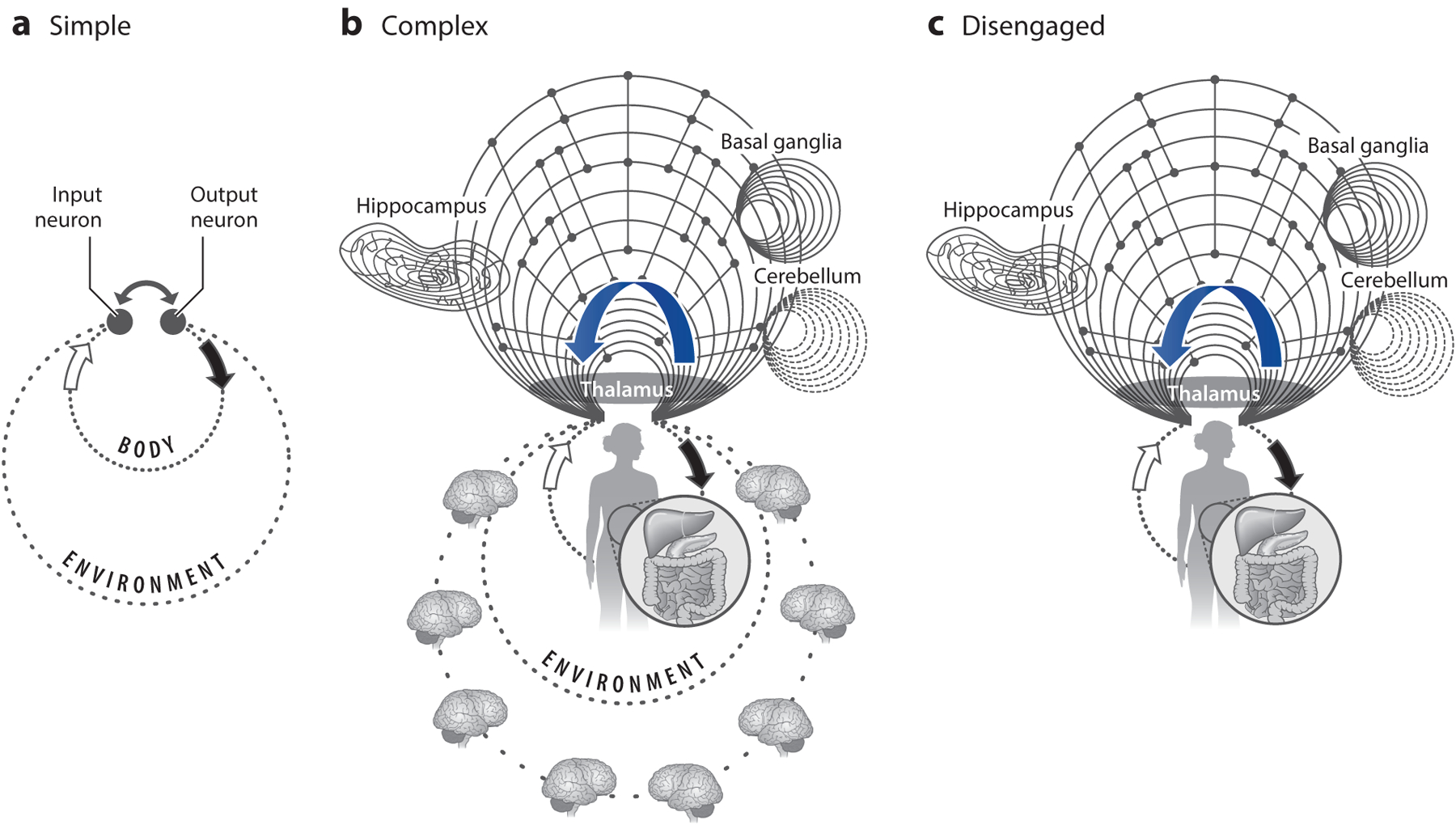
Cognition is environment-disengaged action. During the course of natural selection, organisms adapt to the unique ecological niches in which they live and learn to predict the likely outcomes of their actions in that niche. (*a*) Early organisms with a small nervous system are good at predicting their actions in a relatively constant environmental niche and at a short timescale. (*b*) As brain complexity increases, more intricate connections and neuronal computation insert themselves between motor outputs and sensory inputs. This investment enables the prediction of planned actions in more complex and changing environments and at lengthy timescales far in the future. The curved blue arrow represents corollary discharge from action circuits to sensory and higher-order structures. The many loops work interdependently. Changing the feedback strengths in one loop may affect several other loops. (*c*) More sophisticated brains organize themselves to allow computations to continue even when the animal’s actions come to a halt and sensory inputs vanish temporarily (disengaged or cognitive operation). Adaptive behavior is the result of the continuous interaction between the nervous system, the body, and the environment, each of which has rich and highly structured dynamics. Figure adapted with permission from Buzsáki (2013).

The only grounding source available for neurons in the brain about a detected change derives from the activity of the action circuits. Circuits that send outputs to the body, including hormone-releasing circuits, also send a copy of their actions to large parts of the brain, including sensory areas (Figure 1). These copies, known as corollary discharges [or reafferenz (Sperry 1950, von Holst & Mittelstaedt 1950)], inform the rest of the brain about the action initiated. This corollary message provides the second opinion sensory circuits need for grounding—a signaling mechanism indicating that “my own action is the agent of change” (Buzsáki 2019, p. 62). Recently, internal signaling has been referred to as a forward model (Wolpert et al. 1995) or predictive coding (Rao & Ballard 1999), but these terms refer to the same principles and perhaps related mechanisms. We suggest that akin to an efference copy for muscle activation, there is also an efference copy for an organism’s autonomic, endocrine, and immune state, allowing the organism to detect and coordinate complex internal action sequences in preparation for the future, as referred to by the general term allostasis (Sterling & Eyer 1988).

In summary, the brain interacts with its environmental niche through organs of the body. Due to the coevolution of the nervous system and periphery, there is extensive matching between their properties. We hypothesize that inputs from sensors alone are not sufficient for the brain to learn about the organism’s ecosystem and guide its future actions. Instead, the brain’s actuators— skeletal, autonomic, and endocrine systems together—are essential for attributing significance and meaning to inputs impinging on the organism.

### Primacy of Action and Internal Senses: Body Teaches Brain

Starting from an evolutionary perspective on brain function, it becomes clear that the brain did not evolve to become an information-processing device (Marr 1982) to perceive and then act. Survival is the organism’s most important business (LeDoux 2022), which depends on maintaining a state of relative constancy of its internal environment and operations (homeostasis).

The primacy of action and internal senses view is supported by phylogenetics. Early invertebrates were inconspicuous suspension feeders, capturing and ingesting floating particles by essentially running into them (Striedter & Northcutt 2020). Example organisms are jellyfish, brittle stars, many annelid worms, and other cnidarians. When food is abundant there is no need for sensors to detect them; direct interception and diffusional deposition are sufficient. During the Cambrian period, early vertebrates evolved pharyngeal muscles that allowed them to pump water through their pharynx, thereby gaining the ability to modulate the rate at which animals could obtain food. A hypothetical algorithm to optimize this early action-only system requires only a single internal “representation” of the body’s energy store:


if energy_deficit = = True:
swim( ) *# accelerated movement increases the probability of direct contact with food*
else: rest( ). 


This algorithm is likely present in all animals and requires no external sensory information.

Accompanied by the evolution of vascularized gills, the increased water flow also facilitated gas exchange, enabling early vertebrates to evolve movable jaws and larger bodies and to become more powerful swimmers. In turn, these changes in the body were followed by development and transformations of the sense organs. Particularly striking was the transformation of a small un-paired photoreceptor organ into a pair of relatively large, image-forming eyes, which allowed more sophisticated vertebrates to see potential food items, landscapes, and predators at a distance. To improve action-perception interactions, they also evolved a third canal to add to the two-canal vestibular apparatus, which helped them stabilize retinal images and become even more efficient swimmers (Striedter & Northcutt 2020). In short, external sensors became advantageous to the organism after the development of internal sensors and action systems, which are required for external sensors to be oriented optimally and respond more effectively to the externally sensed inputs (Llinás 2002). Under the outside-in framework, sensory and motor areas are separated by the decision-making circuits. However, a barrage of recent papers has reported that spontaneous body movements and locomotion exert a profound velocity-dependent effect on neuronal firing rate in a host of classical sensory areas (Niell & Stryker 2010). Discussion of these recent works and examples of bodily supervision of the brain during early ontogenesis are available in the Supplemental Appendix.

## COGNITION FROM ACTION

A main reason why the body plays an undeservedly low role in the studies of cognition is that in our everyday experience we do not need to move, walk, or even adjust the sensors to think, recall, plan, imagine, or feel. Accordingly, many aspects of cognition can be studied in immobile subjects in whom the relationship between the consequences of action has already been calibrated by experience. While this argument is true, it does not justify the outside-in model, nor does it diminish the role of action circuits in the brain.

An alternative, evolutionary, hypothesis is that cognition is an exaptation and expansion of the circuits and algorithms serving bodily functions. As organisms continued to evolve more complicated bodies, more precise control and coordination of internal bodily functions were also required. Early neuronal circuits developed a symbiosis with the body to support the body’s fundamental functions, such as ion equilibrium homeostasis, energy management, metabolism, respiration, eating, drinking, excretion, temperature regulation, movement, sensing and responsiveness, reproduction, and sleep (Cisek & Hayden 2022, LeDoux 2022). Although many of these survival mechanisms exist even in organisms lacking a nervous system, neuronal circuits interacting with body functions perfected them, enhancing the organism’s prosperity.

It is conceivable that the earliest survival circuits engaged stimulus-response reflexes and input-initiated homeostasis. However, such hardwired implementations often fail as system complexity grows (Mitchell 2020). Instead, the flexible production of sequences of actions offers a better solution (Åström et al. 2001). As bodies became more complicated, more sophisticated neural algorithms were needed to keep track of the spatiotemporal evolution of action sequences and their internal consequences. We propose that early neural circuits (e.g., the primordial hippocampus) allowed for the effective prediction of internal states through flexible action sequences and the exploration of body space to construct action maps. These prediction circuits allowed for a more efficient exploration of the organism’s niche. As we discuss below, spatial navigation is not simply a series of responses to environmental cues but an internally organized neuronal sequence operation that can be matched to external landmarks. In turn, when the internally organized neuronal sequences disengage from the body actuators, the ensuing fictive or virtual navigation computed by these same circuits can be equated with ideas of memory, planning, and imagination (Figure 1). A feature of this hypothesized exaptation is that even the most complex cognitive and emotional operations keep their dependence on the action repertoire of the organism. Thinking thus may be conceptualized as time-deferred action in the body-disengaged brain (Buzsáki 2019). The utility of thought can be evaluated only if the content of thought is acted out sometime in the future. In summary, cognition is prospection, inducing current or time-deferred actions to acquire a desired goal. Below, we discuss these seemingly disjunctive, yet likely related, brain operations and entertain the possibility that brain-body partnerships evolved initially for internal regulation of complex bodies and later gave rise to cognitive processes.

### Synchronization of Body-Brain States

**Figure 2:**
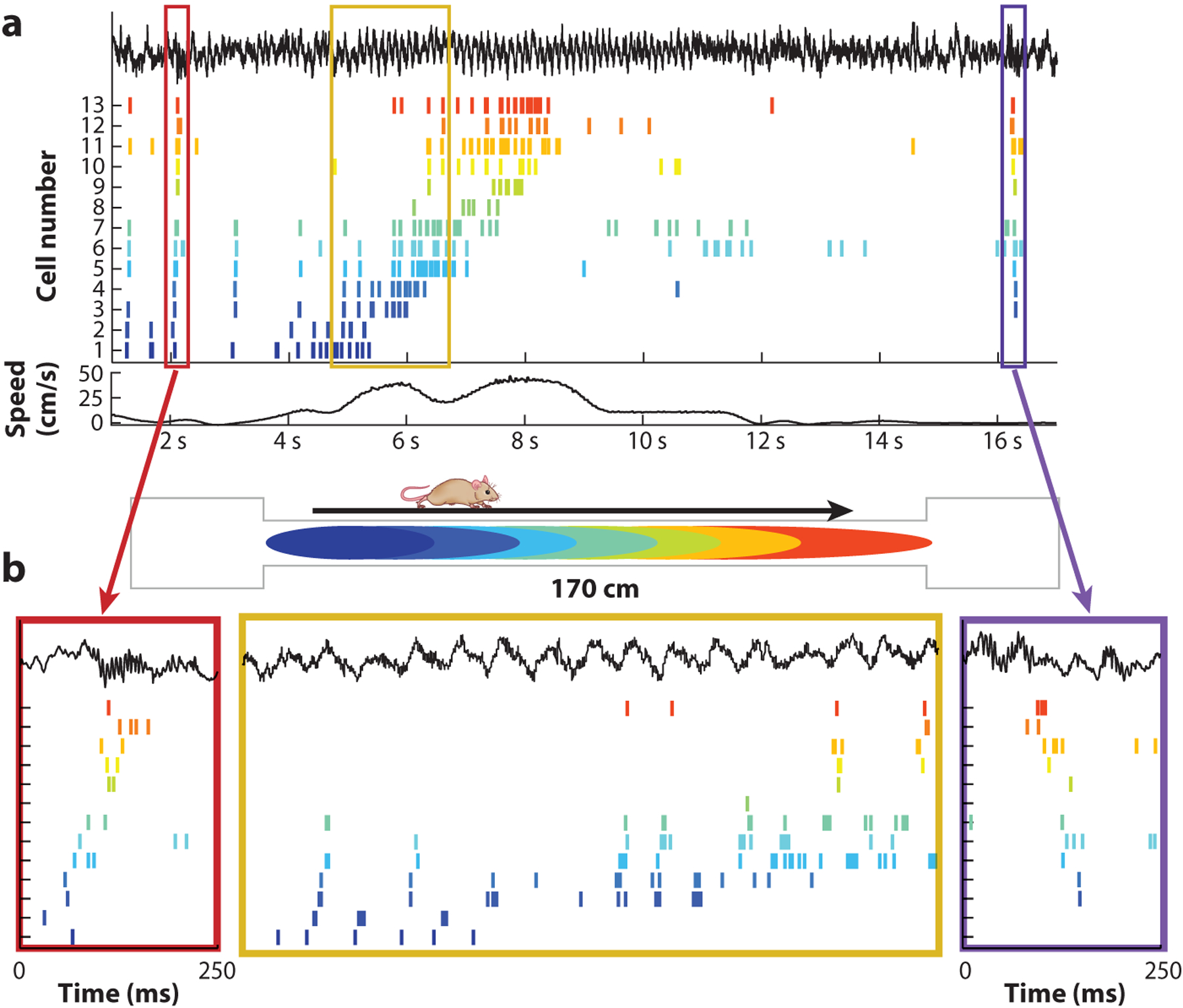
Time compression of neuronal assembly sequences. (*a*) Spike trains of 13 hippocampal neurons (*color ticks*) before, during, and after a single lap. The top black trace illustrates the local field potential; the bottom black line illustrates the locomotion speed of the rat. (*b*) Spike sequences within single theta cycles are compressed versions of the place field activity on the track (2-s segment highlighted in the *yellow box*). These theta sequences gradually shift as the animal moves from left to right down the track. On each end of the track (*red* and *purple boxes*), spiking during ripples reflects forward and reverse replay of the sequences on the track, respectively. Figure adapted from Diba & Buzsáki (2007).

If we consider the simple swim-or-rest algorithm described above, an organism can get quite far in its niche with essentially two action states and no external sensory inputs (Striedter & Northcutt 2020) (Table 1). In animals with a nervous system, this bistable algorithm has been mechanistically—and bidirectionally—coupled to actions of the body throughout evolutionary history and can be observed when taking physiological measurements from essentially any body part (Table 1). Brain states in vertebrates also fall into dichotomous categories and correspond roughly to what early behavioral research referred to as preparative and consummatory (or terminal) classes (Craik & Lockhart 1972). In mammals, these two fundamental brain states can be readily identified by basic electrophysiological monitoring of various brain structures such as the hippocampus (Vanderwolf 1969) (Figure 2; Table 1) or neocortex (Vanderwolf & Stewart 1986). They are also referred to as voluntary and nonvoluntary or conscious and nonconscious brain states (Vanderwolf 1969). Switching between these states is triggered or at least correlated with high and low release of subcortical neuromodulators, respectively (McCormick et al. 2020). Consummatory behaviors include feeding and drinking, resting and its extreme form, sleep (Table 1). Thus, all behaviors can be considered as part of a sequence of action-rest transitions.

The consummatory brain state is an obvious form of disengagement when interaction with the environment is reduced. Brain-body interactions persist but are qualitatively different. Spike activity of neurons in many structures continues, albeit in a different format. During such states, sharp-wave ripple (SPW-R) events are the dominant activity form of the hippocampus. During SPW-Rs, a large fraction of neurons fire together in a highly synchronized manner (Buzsáki 2015).

The participation and specific patterning of neurons within SPW-Rs have been extensively studied in relation to the ambulatory features of previous preparative states (Wilson & McNaughton 1994). However, little is known about the relationship between SPW-Rs and other physiological changes that occur simultaneously during the same consummatory state.

### Multiplexed Reader Mechanisms of Hippocampal Messages

Research on the spike content of theta oscillations and SPW-Rs, and related coordinated activity in cortical areas (Frankland & Bontempi 2005), has focused mainly on the cognitive and mnemonic aspects of behavior during the past decades with only tangential references to their relevance to bodily functions. Below, we highlight work suggesting that the primordial algorithm of hippocampal circuits was to regulate bodily functions, providing the computational backbone on which mnemonic processes would later be placed.

**Figure 3:**
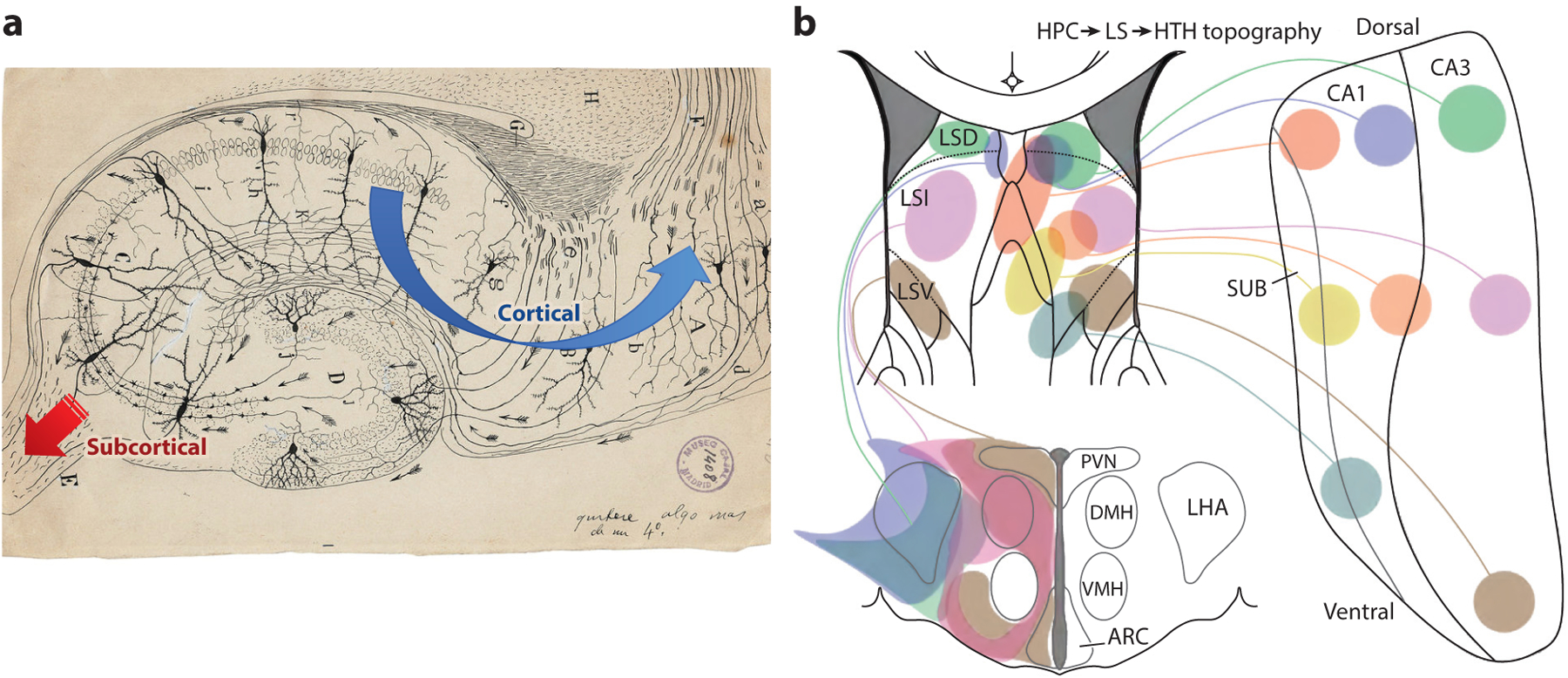
Ramón y Cajal’s error and importance. (*a*) In Ramón y Cajal’s original depiction of the direction of neuronal communication, all his arrows point toward the fimbria, the main subcortical output of the hippocampus, without any indication that hippocampal computations in response to cortical inputs are returned to the cortex. Per his scheme, the hippocampus collects cortical inputs and funnels its condensed version to subcortex. This depiction gave rise to the suggestion that the hippocampus is part of the emotional brain. Over the past decades, the hippocampal-neocortical dialog stepped to the front at the expense of the subcortical output. Image from Cajal Institute (public domain). (*b*) Yet, subcortical projections, including the lateral septum-hypothalamus circuits, are important aspects of hippocampal operation. Abbreviations: ARC, arcuate nucleus; CA, cornu Ammonis; DMH, dorsomedial hypothalamus; HPC, hippocampus; HTH, hypothalamus; LHA, lateral hypothalamus; LS, lateral septum; LSD, dorsal lateral septum; LSI, intermediate lateral septum; LSV, ventrolateral septum; PVN, paraventricular nucleus; SUB, subiculum; VMH, ventromedial hypothalamus.

The anatomical organization of the hippocampal outputs, that is, subcortical and cortical projections, hints to a simultaneous dual functional role of hippocampal physiology (Figure 3; Supplemental Appendix). From an evolutionary standpoint, this organization is illuminating since SPW-Rs are present in the hippocampal homologs of lizards and fish (Shein-Idelson et al. 2016, Vargas et al. 2012), animals with a limited cortex-like reader to decipher the spike content of SPW-R messages. This lineage suggests that SPW-Rs, and hippocampal processes in general, evolved prior to the development and expansion of neocortex in mammals (Cisek & Hayden 2022). Thus, we hypothesize that hippocampal circuits evolved first to support important bodily functions, described below, and were later exapted for use in the hippocampo-neocortical dialog thought to support episodic memory.

From a sender-receiver viewpoint, the SPW-R can be characterized as a multiplexed signal rather than as a unitary broadcast of mnemonic content, and the specificity of reading depends on a transformation function (Swanson et al. 2020). The strongly divergent (fan-out) hippocampal-entorhinal-retrosplenial cortex outputs allow the hippocampus to communicate with virtually all parts of the neocortex. These reader structures, with their many-fold more neurons, are able to perform a decompression computation and therefore extract multiple unique features from the subspace of hippocampus-wide sequential firing (Swanson et al. 2020).

This cortex-wide fan-out communication stands in sharp contrast with a second, and until recently relatively neglected, job of the hippocampal system, which is to affect its subcortical partners (Figure 3). These subcortical structures, such as the lateral septum and hypothalamus, receive strongly convergent inputs from the hippocampal system (Swanson et al. 2020). As may be expected from such a fan-in architecture, these nuclei with relatively few neurons but strongly convergent inputs have a limited ability to read out the spiking content of upstream messages and respond mainly to the magnitude of SPW-R-associated population synchrony, but less so to their informational content (Tingley & Buzsáki 2020), thus performing a type of compression computation on hippocampal activity patterns. This dual hippocampal output implies a dual—body control and cognitive—function of hippocampal operation. Given that the carriers of these output messages are collaterals of largely the same pyramidal neurons, the hypothesis that cognitive operations are inseparably tied to body control is further supported.

## ROLE OF THE HIPPOCAMPUS IN BODY-BRAIN-BODY

### REGULATORY LOOPS

#### Hippocampus Modulates Body States

Since SPW-Rs in the hippocampus dominate nonREM, one should ask whether and how SPW-Rs affect body functions. Sleep is not only prime time for memory (Buzsáki 2015) but also intricately connected to hormonal and metabolic homeostasis in the body and is important in boosting immune-defense mechanisms. It is a well-choreographed internally organized activity with orderly sequences of REM, nonREM, and microarousal stages (Fuller et al. 2006, Watson et al. 2016). Sleep is the default state of brain operations and as such faithfully mirrors the status of brain networks in both health and disease. Although sleep and the circadian rhythm are dissociable processes, under physiological circumstances virtually all functions that they regulate occur synergistically in both the body and the brain (Antle & Silver 2005).

During sleep, the brain exerts its main effects on the body through two axes, that is, the autonomic nervous system (ANS) and the endocrine system. While these peripheral systems work relatively autonomously to supervise heart rate, blood pressure, respiration, digestion, metabolism, temperature regulation, and reproduction (Hubbard 1974), they both influence and, in turn, are influenced by brain activity (Karemaker 2017, Wehrwein et al. 2016, Yao et al. 2022), providing an action-feedback loop. Human imaging studies have shown a high correlation between peripheral autonomic function and brain activity as revealed by functional MRI (Patterson et al. 2002). NonREM and REM states provide a division of labor in mobilizing the parasympathetic and sympathetic components of ANS, respectively, and in regulating the release of various hormones. Release of growth hormone is largely confined to the deepest nonREM state during the first part of the night, dominated by high-power cortical delta waves and hippocampal SPW-Rs, while pituitary-adrenal activity and cortisol levels are increased during the second half of the night, dominated by REM episodes (Alford et al. 1973, Plihal & Born 1999). Hippocampal damage has a profound effect on the release of peripheral corticosteroids in rats, cats, monkeys, and humans (Bohus 1961, Rubin et al. 1967, Slusher & Hyde 1961). Its electrical stimulation elicits various hormonal responses, including changes of glucocorticoids, glucose, and insulin concentrations in the blood stream (Anthony et al. 2014, Seto et al. 1983). However, studying the direct relationship between circulating hormone levels and brain patterns has been hampered by the lack of fast monitoring methods of endocrine dynamics. Recently, the availability of a fast detection technique of peripheral glucose levels offered an indirect window for studying such correlations because the organs most directly related to glucose homeostasis—the liver, pancreas, and adrenal gland— receive redundant signals from the ANS and hypothalamic-pituitary-adrenal axis (Bornstein 1999, Shimazu 1987).

#### Hippocampal Sharp-Wave Ripples Reduce Peripheral Glucose Levels

Large epidemiological studies document a strong relationship between sleep disturbance, obesity, and diabetes (Anothaisintawee et al. 2016). Even moderate sleep loss over time has been linked to increased risk of obesity and diabetes (Kawakami et al. 2004). Selective nonREM perturbation in healthy subjects disrupts insulin sensitivity and glucose tolerance to levels that are judged as an increased diabetes risk (Tasali et al. 2008). Insulin sensitivity varies during the circadian cycle, thus food is metabolized differently during waking and sleep (Sharma & Kavuru 2010). Manipulation of hypothalamic neurons containing orexins/hypocretins not only affects sleep but also can induce hyperphagia and obesity (Antunes et al. 2001, González et al. 2016, Hara et al. 2001). In addition, the ability to acquire and recall information varies across day-night hours (Smarr et al. 2014). However, the neuronal network conditions and activity patterns within the brain that may play a role in these hypothesized regulations have remained undisclosed.

**Figure 4:**
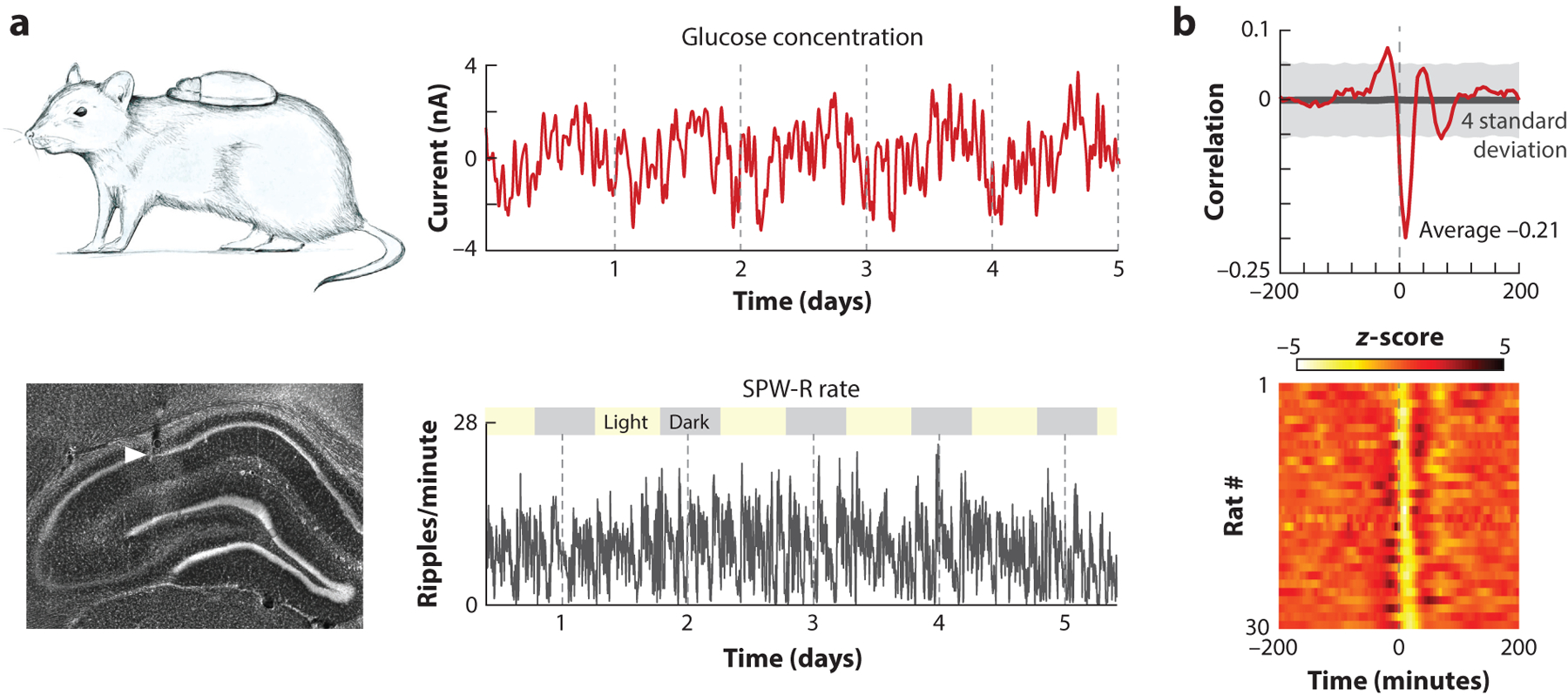
Hippocampal sharp-wave ripples (SPW-Rs) decrease glucose levels in the body. (*a*, *top*) Using a continuous glucose monitor revealed multiple timescale fluctuations of interstitial glucose levels (days, hours, minutes). (*Bottom*) SPW-Rs from CA1 of the hippocampus also showed multiple timescale fluctuations. (*b*, *top*) Normalized cross-correlogram between SPW-Rs and interstitial glucose concentration changes is shown across 30 rats. (*Bottom*) Individual cross-correlograms for each rat show consistent patterns across animals. Figure adapted from Tingley et al. (2021).

A recent study examined the temporal relationship between the occurrence of SPW-Rs and fluctuations of interstitial glucose levels in the freely behaving rat (Figure 4) (Tingley et al. 2021). High rates of SPW-Rs were associated with future decreases in interstitial glucose concentrations. Clustered SPW-Rs were considerably more effective than isolated events, arguing in favor of a cumulative effect. The glucose level–reducing effect was consistent across all consummatory substates (food intake, quiet wakefulness, and nonREM sleep) and could be replicated with op-togenetically induced hippocampal ripples. Because several thousand ripples occur each night in humans, ripple-induced reduction of glucose levels may be critical for energy homeostasis.

SPW-Rs may exert their metabolic effect on peripheral glucose levels via the direct sympathetic and parasympathetic innervation of the pancreas and liver (Niijima 1986, Shimazu 1987). An alternative, or parallel and redundant, pathway is through the regulation of glucocorticoids, and/or growth hormones, via the hypothalamic-pituitary-adrenal axis. Both glucocorticoids and growth hormones are known to impact insulin release and glucose homeostasis (Kim & Park 2017, Kuo et al. 2015). Both or either of these pathways are likely routed through the lateral septum since pharmacogenetic suppression of their neurons abolished the correlation between SPW-Rs and glucose concentration.

Insulin, the main anabolic hormone to regulate glucose uptake, is not released continuously from the beta cells of the pancreas but in synchronized bursts that recur with periodicities of 4–15 min and 90–120 min (Lang et al. 1979, Pørksen 2002). Importantly, these oscillatory bursts can be triggered or phase-reset by a single stimulus such as carbachol infusion or vagus nerve stimulation (Shigeo et al. 1987, Zhang et al. 2008). Analogously, hippocampal SPW-R bursts may provide the physiological signal for resetting and entraining these pancreatic oscillations. After a new experience, the rate of SPW-Rs during consummatory states increases (Buzsáki 2015), supporting the hypothesis that SPW-Rs affect the relationship between glucose metabolism and memory (Korol & Gold 1998).

#### Hippocampus as an Internal Sensor of Body State

The hippocampal system not only exerts an influence on the peripheral autonomic and endocrine systems but also senses the numerous molecules the body constantly produces, providing the necessary feedback for allostasis. The stress hormone corticosterone, produced by the adrenal gland, is a particularly interesting and well-studied example of a brain-body-brain regulatory loop. It is selectively transported into the hippocampus from the bloodstream (McEwen 2007) where it is required for hippocampal adult neurogenesis. Adrenalectomy leads to near complete loss of dentate granule cells (Sloviter et al. 1989), whereas hippocampal damage abolishes normal corticosterone signaling and appropriate stress responses (Buchanan et al. 2009).

The brain is reliant on glucose as a fuel and orchestrates glucose homeostasis by sensing glucose and modulating peripheral metabolism on the timescale of minutes. While several regulatory checkpoints have evolved to safeguard glucose storage and mobilization, the brain, as an added loop in this homeostatic process, has the unique ability to predict the metabolic needs of the body for future behaviors. Brain circuits work cooperatively with peripheral organs to adjust glucose production, storage, and utilization to establish the biologically defended level of glycemia (Alonge et al. 2021, Mirzadeh et al. 2022, Shimazu 1987). As a key participant in these circuits, the hippocampus undergoes large metabolic fluctuations across consummatory-to-preparatory behavioral transitions (McNay et al. 2001), and both insulin and glucose balance are required for normal functioning of hippocampal circuits (Gold 1986, Pearson-Leary et al. 2018). Insulin-sensitive glucose transporters and insulin receptors are found at high levels in the hippocampal formation (Schulingkamp et al. 2000, Unger et al. 1989), and insulin can be trafficked to these receptors (Banks & Kastin 1998). Taken together, the hippocampal system appears to have privileged access to stress and metabolic states of the body and an ability to bias them (McEwen 2007).

**Figure 5:**
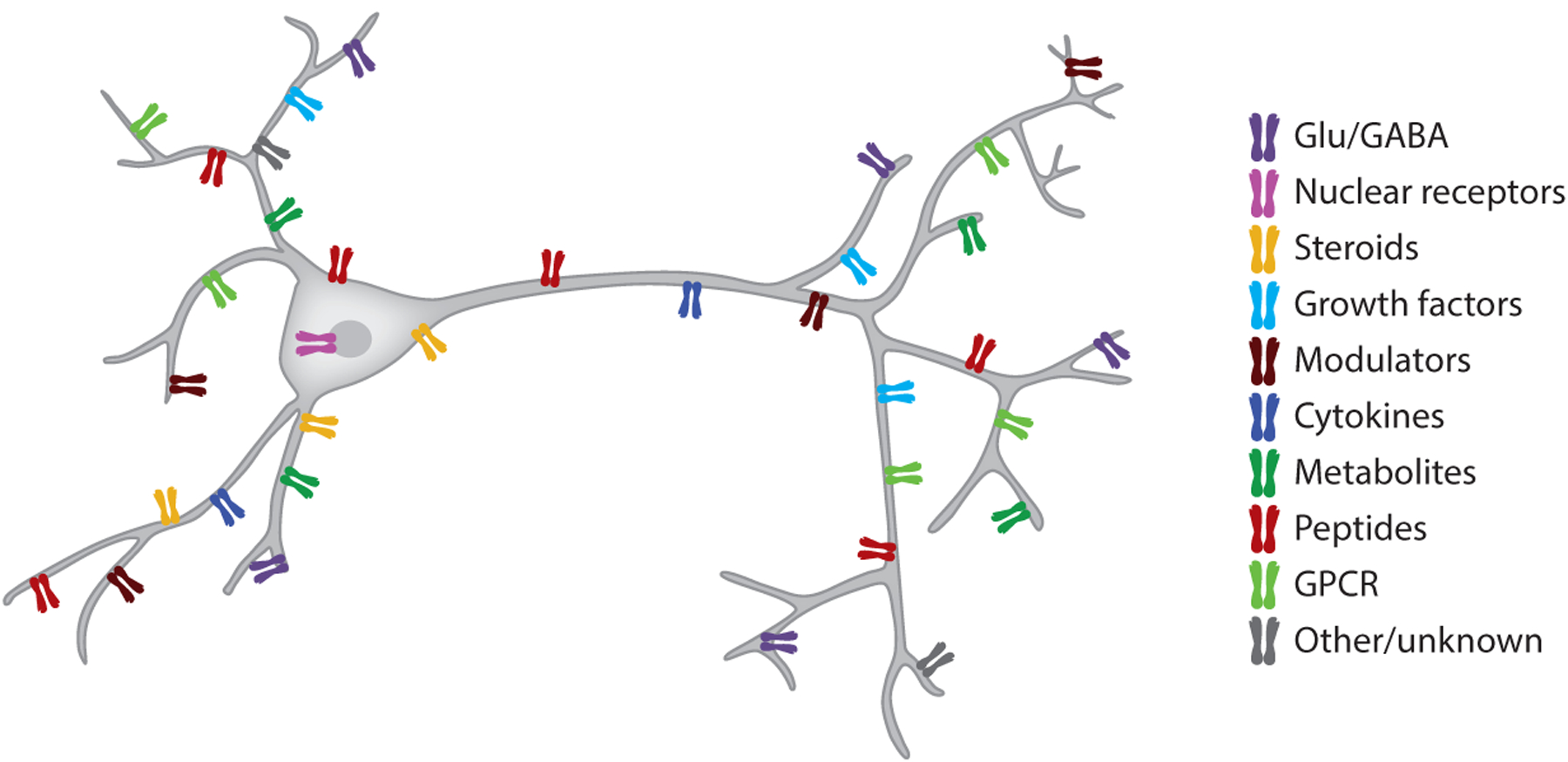
Neurons are controlled by neurotransmitters, neuromodulators, hormones, immune factors, and other body-produced substances. In addition to receptors for GABA, glutamate, and canonical neuromodulators (Ach, DA, 5-HT, NE), the average mouse cortical neuron expresses RNA transcripts for more than 60 receptors for metabolites, hormones, peptides, and immune-related molecules. Abbreviations: 5-HT, serotonin; Ach, acetylcholine; DA, dopamine; GPCR, G protein–coupled receptor; NE, norepinephrine.

The high dimensionality of this internal sensor function is well illustrated by the high fraction of neuronal and glial receptors influenced by circulating substances (Lathe 2001). While many veins of neuroscience research conceptualize an average neuron integrating at most a dozen types of signals (i.e., GABA, glutamate, and canonical neuromodulators), a typical hippocampal neuron possesses receptors and channels that allow it to integrate a minimum of 60 unique signals (i.e., metabolites, hormones, temperature) (Lathe 2001, McEwen 2007) (Figure 5). These nonsynaptic neuronal and glial receptors are activated by the many substances produced by the immune system (Besedovsky et al. 1983, Dantzer et al. 2008), peripheral organs, and microbiota-gut-brain signaling axis (Cryan et al. 2019).

The hippocampus and its phylogenetic homologs are especially fit for sensing circulating substances. The hippocampus essentially floats in the lateral ventricle and is an interface with a surface area several orders of magnitude larger than that of the border between the third ventricle hypothalamic nuclei, making hippocampal circuits highly susceptible to modulation by the proteome of cerebrospinal fluid. The implications of integrating such a high-dimensional humoral space into the computations the hippocampus performs remain an exciting future direction of research.

#### Closing the Loop

Overall, the hippocampus appears to play a role in at least two brain-body-brain regulatory control loops, including corticosterone and stress responses and glucose and peripheral metabolism. A technical hurdle for studying whether hippocampal SPW-Rs (or other activity patterns) are also participating in these and other regulatory loops relating to other metabolites, hormones, or signaling molecules is our ability to measure such substances in vivo at the speed of neuronal actions. The main hormonal effector of the brain, the hypothalamus-pituitary gland, produces a dozen hormones and releases them into the bloodstream (McEwen 2007). Pioneering work has begun to describe similar control loops that involve ghrelin (Hsu et al. 2015, Kanoski & Grill 2017, Kim et al. 2017), leptin (Harvey et al. 2006, O’Malley et al. 2007), and reproductive hormones (Taubøll et al. 2015). There are other feedback loops, including the many substances produced by the immune system (Besedovsky et al. 1983), organs of the body (Friedman & Halaas 1998), and microbiota-gut-brain signaling axis (Morais et al. 2021) (Figure 5). The specific neuronal network patterns on which they exert their effects, and that regulate their release, will need to be identified.

## EXAPTATION OF MEMORY FROM NAVIGATION

In addition to its role in endocrine body-brain-body control—perhaps the first function in evolution—the hippocampal system is also involved in navigation and memory. When a rodent walks through a maze, distinct assemblies of place cells (O’Keefe & Nadel 1978) become active in the hippocampus. Collectively, these neurons, along with their grid cell partners of the medial entorhinal cortex (Hafting et al. 2005), are believed to form a template or a map of the environment. One possible interpretation of the relationship between position and neuronal firing is that the continually changing sensory inputs from the environment control the activity of neurons (O’Keefe & Burgess 1996). However, the hypothetical map is only an aid for navigation to provide static positional information (such as a GPS) in an allocentric reference frame from the relationships among landmarks. This neuronal map is scalable, and the metric needed for the estimation of distances between landmarks in a concrete environment arises from a second mechanism, known as self-referenced (or egocentric) navigation (McNaughton et al. 1996). The essential components of this self-referenced navigation are the initial reference position, head direction, locomotion speed, and elapsed time from which the vectorial aspects of travel can be calculated by integrating exafferentation from motion-related body reafferentation and visual and haptic flow [known as path integration (McNaughton et al. 1996)]. An alternative explanation is that neuronal sequences in the navigation system are internally generated, even in an inexperienced brain. The abstract concepts of space and locations thus may arise from matching these preexisting or preconfigured sequences to action sequences of travel paths (Buzsáki & Tingley 2018, Dragoi & Tonegawa 2011, Huszár et al. 2022, Pastalkova et al. 2008). Only this latter, self-organized mechanism can link the hippocampal system to its other function: memory.

If internally organized sequences underlie spatial navigation, it should not come as a surprise that there is a clear parallel between egocentric navigation and episodic memory, on the one hand, and map-based navigation and semantic memory, on the other (Buzsáki & Moser 2013). Self-organized neuronal sequences, navigation, and personal episodes are vectorial trajectories that unfold in space-time (i.e., a segment with distance and duration). The self moving through space is a history or episode with both spatial and temporal adjacency relationships (Tulving 2002). On the other hand, semantic memory is a frozen subspace of intersecting multiple episodes with disconnected spatial-temporal history (Buzsáki et al. 2022). At the neuronal level, semantic memory may correspond to omnidirectional, context-free place cells that determine each place in the environment explicitly, just as the firing of semantic or concept cells in humans responds to both observed and recalled specific objects or persons (Quiroga et al. 2005). A fundamental difference is that mental navigation does not depend on immediate environmental or body-exafference cues. It has been hypothesized that neuronal mechanisms, which initially depended on external cues in simple organisms, have become internalized (Buzsáki 2019) in more complex brains so that self-organized neuronal activity can maintain neuronal trajectories. Without external constraints, disengaged brain processing can create an internalized virtual world and generate new knowledge through vicarious or imagined experience, tested against preexisting and stored knowledge (Buzsáki 2019). Reframing cognition as continuous with navigation has the advantage of understanding how memory and thought could form positionally via nested regularities in the relations encountered as the body navigates.

We should emphasize, though, that the hypothesized evolutionary continuity of navigation and memory does not imply that these are distinct mechanisms that developed sequentially. Instead, the mechanisms are strongly intertwined, and the degree to which they dominate depends on the level of brain complexity and the availability of external cues. More complex brains can rapidly acquire multiple representations, hold them as separate over longer timescales, and use them flexibly even in degraded conditions (Eichenbaum 2004). Yet, the fundamental rules in simple and more developed brains remain the same. The hippocampus-hypothalamus-body axis represents the action arm of a homeostatic loop, with the body-to-hippocampus signaling as the return arm. This loop is allostatic, preparing the body for expected future encounters with the animal’s niche. Similarly, long-term memory (past) and attention/working memory (present) enable the brain to imagine/plan efficient actions within the organism’s ecological environment (future). Action outcomes then become memories, creating a memory-plan-memory loop.

In summary, the hippocampal system may have undergone two exaptation processes during evolution. Primordial neural circuits that initially evolved to coordinate sequential events in the body (allostasis) were co-opted to anticipate and incorporate the bodily outcomes in physical exploration of the organism’s niche. Second, these same circuits were again co-opted for an internalized processing of sequences of experience (episodic memory). Thus, the hippocampus may perform a singular algorithm that relates its sequential, relational, content-agnostic mechanisms to actions performed by the body or to an internalized version of action sequences (Buzsáki & Tingley 2018). The disengaged mode of self-organized neuronal activity provides access to a virtual world of vicarious or imagined experience and constitutes a gateway to a variety of cognitive processes. Memory is a transmission mechanism gleaned from past experience to guide current and future actions rather than a storage of symbols of world events and facts (Buzsáki et al. 2022). Thus, navigation through either a physical space or alternatively a landscape that exists only in the imagination (i.e., mental time travel and planning ahead for an action) (Tulving 2002) may be accomplished through identical neural mechanisms. These complex computational mechanisms may have evolved from the need to predict future metabolic needs of the body.

## CONCLUSIONS AND OUTLOOK

Throughout evolution, the brain’s constant partner is the body it serves. A critical task for the brain is to coordinate the numerous body functions. These brain functions, originally evolved to regulate and predict metabolic and motor processes in the body, have undergone various levels of exaptation, expanding the same circuit computations to perform environment-disengaged activity in the service of cognition. Because of these exaptation steps, even the most complex operations keep their dependence on the action repertoire of the organism. The unified goal of both simple and complex neuronal circuits is to predict the consequences of their outputs. At the highest level, cognition is prospection, inducing immediate or deferred actions to acquire desired goals. While the metaphor of embodied cognition has been extensively discussed over decades within cognitive science, methods and conceptual thinking in neuroscience have matured only recently to take a fresh look at the embodied mind or, more appropriately, the embodied brain.

The functioning of an organism depends on its ability to maintain stable homeostatic states, such as body temperature and blood sugar levels, within narrowly regulated ranges in the face of constant perturbations from the environment. Similarly to the whole organism, maintaining a physiological self-organized dynamic of brain circuits is essential for its physiological operation. Maintenance of this dynamic (also known as spontaneous activity or internal state) can also be considered a homeostatic process, which is achieved through cooperation with the body. Maintenance of the internal dynamic is a costly operation, as illustrated by the high energy budget of self-organized network activities (Raichle et al. 2001). More than half of emitted spikes by forebrain neurons serve to maintain homeostatic network dynamics (Levenstein et al. 2022), and most of the remaining, temporally coordinated, spiking across neurons also contributes to internally generated oscillations and assisting homeostatic body functions. Only a small fraction of neurons respond to external stimuli or control body actuators. Thus, sensing and associating environmental stimuli mobilize only a small fraction of neurons at any given time. This responding mode is energetically cheap and secondary compared to sustaining the perpetual internal operations.

Our review illustrated several homeostatic loops within the brain and between brain and body. Even for a seemingly unitary function, for example, glucose regulation, multiple loops act cooperatively. A main issue that remains to be resolved is the interactions among the many loops. It is unlikely that separate homeostatic loops work in isolation. The alternative is that we are dealing with a tangled web of loops; thus, understanding any single homeostatic regulatory loop makes sense only when viewed in the context of related regulation mechanisms. For example, energy, temperature, orthostatic blood pressure, food-seeking motor activity, sleep, and memory appear distinct and are typically investigated in different laboratories, yet changing feedback strengths in one loop may affect several other loops (Figure 1). Multiple substances of the body may converge on the same brain effector mechanisms. Conversely, the same substance/input may differentially activate brain circuits, depending on the affordances available for the organism at different occasions in the environment. Recognizing the interdependence of the many brain-body interactions and, possibly, their hierarchical or nested relationships is a key step in disentangling their specific contributions in such multivariate loops (Northoff 2018). The phrase “internal state” may reflect the relationship between an arbitrarily selected loop and all other simultaneously active homeostatic processes. Thus, the often-used term “region of interest” is hard to justify in most experiments due to the entanglement of many interactive loops. It is of foremost importance that we do not hastily attribute cognitive labels to brain recordings that simply reflect unobserved but recordable activity of body status. Achieving such rigor will require development of novel techniques. Tool development in past decades led to great improvements in recording large population of neurons, which we refer to as depth. In contrast to the ever-increasing depth of observability, the breadth of observations typically made in an experiment has not progressed in nearly 100 years and remains focused on merely a handful of unique types of signals (see notes in the Supplemental Appendix). We acknowledge that this extended task poses added difficulties for an experimentalist. Yet, it is hard to identify an alternate path, and in the long haul, it may save us from false conclusions.

Our review aimed to point out the need and advantages for an evolutionary approach to studying cognition, as opposed to the traditional mind-centric practice in neuroscience. This evolutionary continuity view is expected to ease the tension between works performed on humans and other animals and different systems in the same brains and emphasize the need for comparative studies from simple to more complex organisms. An important step in this direction is the recognition that a primary function of the brain is to serve its body and assist with the survival and prosperity of the organism in its appropriate ethological niche, rather than continue the work in the still-popular perceive-sort-prioritize-decide-act framework. The brain needs the body not only to fuel its energetically expensive computation but also as an essential partner in a symbiotic process. The body is for the brain as the water is for the fish, inseparable to the point that we are largely unaware of its role in our cognitive experiences. And if we take the third-person view of the experienced older fish, we may start seeing the value of the body as the primary teacher of the brain.

## Supplementary Material

NIHMS1955133 Supplemental Material

## Figures and Tables

**Table 1:** Competing fundamental brain-body states

Area	Preparative (voluntary)	Consummatory (nonvoluntary)
**Brain**		
Neocortex	Desynchronized	Slow waves
Hippocampus	Theta	LIA/SPW-Rs
Subcortical neuromodulators	↑ ACH	↓ ACH
	↑ NE	↓ NE
	↑ 5-HT	↓ 5-HT
	↑ HA	↓ HA
**Body**		
Musculoskeletal system	Muscle contraction, locomotion	Rest, eating, drinking, urination, defecation, orgasm, sleep
HPA/HPS-axis	↑ CORT	↓ CORT^a^
	↓ GH	↑ GH
Energy usage	High	Less (but differential)
Liver	Gluconeogenesis (and kidneys), glycogenolysis	Glycogenesis
Pancreas	Glucagon release	Insulin release
Metabolic/body programs	↑ Body temperature	Digestion
	↑ Brain temperature	↓ Body temperature
		↓ Brain temperature
Autonomic nervous system	REM^a,b^	NonREM
	↑ Sympathetic	↑ Parasympathetic
Immune system	Immune compromised/suppressed	Enhanced

a Salivation during sympathetic activation, pupil dilates, and heartbeat increases.

b From a purely physiological perspective, REM versus nonREM divisions correspond to preparatory and consummatory states.

Abbreviations: 5-HT, serotonin; ACH, acetylcholine; CORT, corticosterone; GH, growth hormone; HA, histamine; HPA, hypothalamic-pituitary-adrenal axis; HPS, hypothalamic-pituitary-somatotropic axis; LIA, large-amplitude, irregular activity; NE, norepinephrine; REM, rapid eye movement; SPW-R, sharp-wave ripple.
