# Supplementary material for: Cognition from the Body-Brain Partnership: Exaptation of Memory
Source: Annu Rev Neurosci. Author manuscript; Available in PMC 2024 Jan 17. (PMC10793243; doi:10.1146/annurev-neuro-101222-110632)
Supplement: NIHMS1955133 Supplemental Material [file NIHMS1955133-supplement-NIHMS1955133_Supplemental_Material.pdf]

Supplemental Material: Annu. Rev. Neurosci. 2023. 46:191-210
https://doi.org/10.1146/annurev-neuro-101222-110632
Cognition from the Body-Brain Partnership: Exaptation of Memory
Buzsáki and Tingley

Cognition from Body-Brain Partnership: Exaptation of Memory

Supplemental Text, Figures and Supplemental References

(references of the main text are not re-listed in the Suppl. Material)

INTRODUCTION

Note 1. The brain is an integral part of the body. For the ease of description only, we refer to brain-body relationship as if the body corresponded to all other organs but the brain. Organism is a term related to the body, referring to individual forms of life in bacteria, protists, fungi, plants and animals.

Note 2. Embodied cognition has weak and strong formulations. The weak view states that the body exerts a significant and measurable influence on cognitive processing, implying that failing to monitor changes in the body cannot fully account for cognition. The strong formulation goes further suggesting that the body acts as a (partial) realizer of cognitive processing that is distributed across neural and non-neural entities (Varga and Heck, 2017).

Note 3. While the reductionist principle of *divide et impera* has been repeatedly shown to be an efficient strategy for describing and understanding many aspects of science, it has had limited success in cognitive neuroscience. From this viewpoint, it is presumed that organs of the body—including the brain—have distinct stand-alone functions. While this may appear to be true, there is no bodily organ capable of survival without most other components of the body. Thus, each 'stand-alone function' typically serves the entire body. The brain is no exception, it is useless without the body it evolved to serve, and in turn which it regulates (Varela et al., 1991).

Agre, P.E., 1997. Computation and Human Experience, Illustrated edition. ed. Cambridge University Press, Cambridge ; New York.

Ahissar, E., Assa, E., 2016. Perception as a closed-loop convergence process [WWW Document]. eLife. https://doi.org/10.7554/eLife.12830

Allen, W.E., Chen, M.Z., Pichamoorthy, N., Tien, R.H., Pachitariu, M., Luo, L., Deisseroth, K., 2019. Thirst regulates motivated behavior through modulation of brainwide neural population dynamics. Science 364, 253.

Anderson, M.L., 2003. Embodied Cognition: A field guide. Artif. Intell. 149, 91–130
Andres, M., Olivier, E., & Badets, A. (2008). Actions, words, and numbers: A motor contribution to semantic processing? Current Directions in Psychological Science, 17(5), 313–317.

Barsalou, L. W. (2008). Grounded cognition. Annual Review of Psychology, 59, 617–645.

Brooks, R. (1999). Cambrian Intelligence. Cambridge, Mass.: MIT Press

Buchanan B (2008). Onto-ethologies: The Animal Environments of Uexküll, Heidegger, Merleau-ponty, and Deleuze. SUNY Press.

Clancey, W.J., 1997. Situated cognition: On human knowledge and computer representations, Situated cognition: On human knowledge and computer representations. Cambridge University Press, New York, NY, US.

Clark A. Surfing Uncertainty: Prediction, Action, and the Embodied Mind. *Oxford University Press, 2016.*

Clark, A. (1996) Being There: Putting Brain, Body and World Together Again, MIT Press

Clark, A. (1999). An embodied cognitive science? Trends in Cognitive Sciences, 3, 345–351.

Dourish, P., 2001. Where the Action Is: The Foundations of Embodied Interaction. MIT Press.
Freeman, R.B., 1969. The Senses Considered as Perceptual Systems. James J. Gibson. Q. Rev. Biol. 44, 104–105.

Gallagher, S., 2005. How the Body Shapes the Mind. Oxford University Press.

Gibson, J. (1979). The Ecological Approach to Visual Perception. Boston: Houghton-Mifflin.

Goldin-Meadow, S., & Wagner, S. M. (2005). How our hands help us learn. Trends in Cognitive Sciences, 9(5), 234–241.

Haggard, P., 2017. Sense of agency in the human brain. Nat. Rev. Neurosci. 18, 196–207.

Hegarty, M. (2004). Mechanical reasoning by mental simulation. Trends in Cognitive Sciences, 8(6), 280–285.

Jeannerod, M., 2003. The mechanism of self-recognition in humans. Behav. Brain Res. 142, 1–15.

Kaas, J.H., 2012. The evolution of neocortex in primates. Prog. Brain Res. 195, 91–102.

Kaas, J.H., 2013. The evolution of brains from early mammals to humans. WIREs Cogn. Sci. 4, 33–45.

Lakoff, G., Johnson, M., 1999. Philosophy in the Flesh: the Embodied Mind & its Challenge to Western Thought. Basic Books, New York.

Laurent G, Wolf F, Carr C. Editorial overview: Evolution of brains and computation. Curr Opin Neurobiol. 2021 Dec;71:iii-viii.

Musall, S., Kaufman, M.T., Juavinett, A.L., Gluf, S., Churchland, A.K., 2019. Single-trial neural dynamics are dominated by richly varied movements. Nat. Neurosci. 22, 1677–1686.

Nöe, A., 2006. Action in Perception. MIT Press.

Pfeifer, R., Scheier, C., 2001. Understanding Intelligence. MIT Press.

Pulvermuller, F. (2005). Brain mechanisms linking language and action. Nature Reviews Neuroscience, 6(7), 576–582.

Pulvermuller, F., & Fadiga, L. (2010). Active perception: Sensorimotor circuits as a cortical basis for language. Nature Reviews Neuroscience, 11(5), 351–360.

Rubin, D. C. (2006). The basic-systems model of episodic memory. Perspectives on Psychological Science, 1(4), 277–311.

Siegle, J.H., Jia, X., Durand, S., Gale, S., Bennett, C., Graddis, N., Heller, G., Ramirez, T.K., Choi, H., Luviano, J.A., Groblewski, P.A., Ahmed, R., Arkhipov, A., Bernard, A., Billeh, Y.N., Brown, D., Buice, M.A., Cain, N., Caldejon, S., Casal, L., Cho, A., Chvilicek, M., Cox, T.C., Dai, K., Denman, D.J., de Vries, S.E.J., Dietzman, R., Esposito, L., Farrell, C., Feng, D., Galbraith, J., Garrett, M., Gelfand, E.C., Hancock, N., Harris, J.A., Howard, R., Hu, B., Hytnen, R., Iyer, R., Jessett, E., Johnson, K., Kato, I., Kiggins, J., Lambert, S., Lecoq, J., Ledochowitsch, P., Lee, J.H., Leon, A., Li, Y., Liang, E., Long, F., Mace, K., Melchior, J., Millman, D., Mollenkopf, T., Nayan, C., Ng, L., Ngo, K., Nguyen, T., Nicovich, P.R., North, K., Ocker, G.K., Ollerenshaw, D., Oliver, M., Pachitariu, M., Perkins, J., Reding, M., Reid, D., Robertson, M., Ronellenfitch, K., Seid, S., Slaughterbeck, C., Stoecklin, M., Sullivan, D., Sutton, B., Swapp, J., Thompson, C., Turner, K., Wakeman, W., Whitesell, J.D., Williams, D., Williford, A., Young, R., Zeng, H., Naylor, S., Phillips, J.W., Reid, R.C., Mihalas, S., Olsen, S.R., Koch, C., 2021. Survey of spiking in the mouse visual system reveals functional hierarchy. Nature 592, 86–92.

Steinmetz NA, Aydin C, Lebedeva A, Okun M, Pachitariu M, Bauza M, Beau M, Bhagat J, Böhm C, Broux M, Chen S, Colonell J, Gardner RJ, Karsh B, Kloosterman F, Kostadinov D, Mora-Lopez C, O'Callaghan J, Park J, Putzeys J, Sauerbrei B, van Daal RJJ, Vollan AZ, Wang S, Welkenhuysen M, Ye Z, Dudman JT, Dutta B, Hantman AW, Harris KD, Lee AK, Moser EI, O'Keefe J, Renart A, Svoboda K, Häusser M, Haesler S, Carandini M, Harris TD. Neuropixels 2.0: A miniaturized high-density probe for stable, long-term brain recordings. Science. 2021 Apr 16;372(6539):eabf4588.

Varela, F.J., Thompson, E., Rosch, E., 1991. The embodied mind: Cognitive science and human experience, The embodied mind: Cognitive science and human experience. The MIT Press, Cambridge, MA, US.

Varga S, Heck DH (2017) Rhythms of the Body, Rhythms of the Brain: Respiration, Neural Oscillations, and Embodied Cognition. Conscious Cogn 56:77–90.

von Uexküll J, A Foray into the Worlds of Animals and Humans: With A Theory of Meaning, translated by Joseph D. O'Neil, Minneapolis/London: University of Minnesota Press, 2010.

von Uexküll J. *Theoretical Biology*, New York: Harcourt, Brace & Co., 1926.

Wheeler, M. (2005). Reconstructing the Cognitive World. Cambridge, Mass.: MIT Press.

Wilson, M., & Knoblich, G. (2005). The case for motor involvement in perceiving conspecifics. Psychological Bulletin, 131(3), 460–473.

Zwaan, R. A. (2004). The immersed experiencer: Toward an embodied theory of language comprehension. Psychology of Learning and Motivation: Advances in Research and Theory, 44(44), 35–62.

MIND-BRAIN-BODY

Note 1. The issue of representation has long been discussed in psychology and cognitive science. According to the ‘‘representationalist’’ view, states of the world are “encoded” or “modeled” or mirrored by the states of the mind. A challenging response was offered by behaviorism, arguing that cognition cannot be separated from the activities of the animal (Watson 1924; Skinner). The declined influence of behaviorism was due mainly to its unjustified belief that cognition and decisions can be understood simply by shaping behavior with *post hoc* credit assignment of reward and punishment. The Skinnerian view was revived recently and became popular under the label of “reinforcement learning” (Sutton and Barto, 2014). Even Skinner’s own students recognized the limitations of reinforcement and emphasized the species-specific preconfigured nature of behavioral responses (Breland and Breland, 1961) and the limitations are well-illustrated by the extensive literature on “autoshaping” (Brown and Jenkins, 1968), arguing that signals and reinforcers simply provide an opportunity for implementation of innate tendencies (Buzsaki, 1982). European ethology, focusing on detailed description of behavior in the animal’s ecological niche was an important antidote for both representationalism and reinforcement learning (Frisch 1953; Lorenz 1949; Tinbergen, 1951;). Ethology and experimental psychology have produced ample demonstrations that both simple and complex organisms are prepared to associate certain events, unprepared to associate others, and contra-prepared to associate still others (Seligman 1970). A related but somewhat independent line of research is exemplified by the Gibsonian ecological psychology (Gibson 1966, 1979), a direct descendent of American naturalism. Ecological psychology advanced the thought that behavior is not for gathering information but to guide actions afforded by the organism’s niche. We perceive the surroundings in order to do things. Embodied cognition and the situated mind (Brooks 1991) movement grew out from ecological psychology but it had very few followers in neuroscience (Chemero 2009; Freeman and Skarda, 1990; Juavinett et al., 2018; Krakauer et al., 2017; Parker et al., 2020).

Note 2. In addition to limiting behavioral observations related to brain activity, head-fixing is deemed extremely stressful by placing the practice in USDA Category E. Rodents are both acrophobic agoraphobic and placing them in virtual and physical open space is aversive and likely results in distorting many brain-behavior correlations (Suppl Fig. 1).

Note 3. “Representation” is a key ingredient of the outside-in framework. What it actually means in terms of brain mechanisms is often not clear. It has some relationship to the idea of the “engram” which refers to a conceived physical embodiment of an event. Representation is fundamentally a relationship between some aspects of the world with its objects and events and brain activity. But whether and how different brains of different animals represent the same thing (the objective “truth”) is rarely asked (Freeman and Skarda, 1990). One can approach “representation” from a soul-less point of view of a machine. The exploration of a navigating robot allows it to detect and register correlations between its particular own movements and sensory inputs from landmarks, such as walls. This is possible only because of the robot’s structured, low-level activity of wall following, which allows it to infer a relationship between its movements and sensor readings (e.g., distances). The exploration unfolds in time, thus landmarks have extension over time. There are no explicit instructions inside the robot that tell it to follow a wall, there is no centralized decision maker and of course there is no subjective representation of walls or landmarks. Landmarks come into being as the result of the robot’s structured activity within a particular environment (George et al., 2021; Henriks-Jansen, 1996). Intelligent behavior of autonomous robots is an emergent property of a machine (an “agent” in the parlance of AI) embedded in an environment with which it continuously interacts.

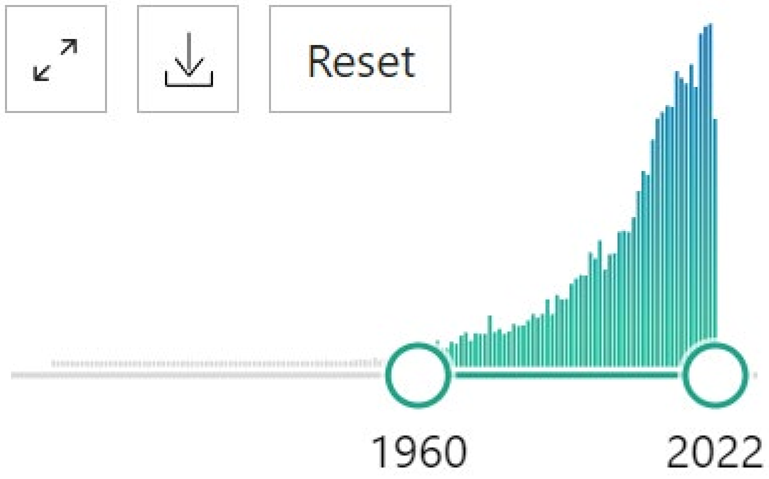

Supplementary Fig. 1. The number of publications with ‘head fixation’ used as a search term (PubMed).

Breland K, Breland M. 1961. The misbehavior of organisms. Am. Psychol. 16(11):681–84

Brown PL, Jenkins HM. 1968. Auto-shaping of the pigeon’s key-peck. J. Exp. Anal. Behav. 11(1):1–8

Buzsáki G. 1982. The “where is it?” reflex: autoshaping the orienting response. J. Exp. Anal. Behav. 37(3):461–84

Chemero A. (2009) Radical Embodied Cognitive Science. A Bradford Book. The MIT Press. Cambridge, MA.

Freeman WJ, Skarda, C.A. (1990) Representations: Who needs them? In: Third Conference, Brain Organization and Memory: Cells, Systems and Circuits. (Eds.) J.L. McGaugh JL, Weinberger NM, Lynch G (eds.). New York, Oxford, Guilford Press.. pp. 375-380.

George D, Rikhye RV, Gothoskar N, Guntupalli JS, Dedieu A, Lázaro-Gredilla M. Clone-structured graph representations enable flexible learning and vicarious evaluation of cognitive maps. Nat Commun. 2021 Apr 22;12(1):2392.

Guide for the Care and Use of Laboratory Animals https://grants.nih.gov/grants/olaw/guide-for-the-care-and-use-of-laboratory-animals.pdf

Hendriks-Jansen H (1996) Catching Ourselves in the Act: Situated Activity, Interactive Emergence, Evolution, and Human Thought. A Bradford Book. The MIT Press.

Juavinett, A.L. et al. (2018) Decision-making behaviors: weighing ethology, complexity, and sensorimotor compatibility. Curr. Opin. Neurobiol. 49, 42–50

Lau, H.C., Passingham, R.E., 2006. Relative blindsight in normal observers and the neural correlate of visual consciousness. Proc. Natl. Acad. Sci. U. S. A. 103, 18763–18768.

Lorenz K (1949/2007). King Solomon's Ring (3rd ed.). London: Routledge.

Parker PRL, Brown MA, Smear MC, Niell CM. Movement-Related Signals in Sensory Areas: Roles in Natural Behavior. Trends Neurosci. 2020 Aug;43(8):581-595.

Seligman ME. 1970. On the generality of the laws of learning. Psychol. Rev. 77(5):406–18.

Skinner BF (1938) The Behavior of Organisms: An Experimental Analysis. Appleton-Century - Crofts.

Sutton RS and Barto AG. 2014. Reinforcement Learning: An Introduction. Second edition. A Bradford Book. The MIT Press.

Tinbergen N (1951) The Study of Instinct. Oxford, Clarendon Press.

Von Frisch (1953) The Dancing Bees: An Account of the Life and Senses of the Honey Bee. Harvest Books, New York.

Watson JB. (1924/1958) Behaviorism (revised ed.). Chicago: University of Chicago Press.

Wilson, R.A., 2004. Boundaries of the Mind: The Individual in the Fragile Sciences - Cognition. Cambridge University Press, Cambridge.

BODY-BRAIN-MIND

Primacy of Action and Internal Senses – Body Teaches Brain

Note 1. *Intrauterine teaching of the brain by the body*. Internally generated activity and outputs are also critical during ontogenetical development (Katz and Shatz 1996). For example, during intrauterine life, the body teaches the brain about the rapidly changing body scheme. Irregular small muscular twitches, limb jerks and whole-body startle responses ("baby kicks") emerge during the latter part of the second trimester of pregnancy. These actions trigger the first organized brain patterns, known as "early spindles" or "delta brush". The topographic distribution of these induced physiological events in the somatosensory areas has a correspondence with the body parts (Khazipov et al., 2004; Milh et al., 2007). Due to the physical constraints of the bones and joints, only a limited fraction of muscle movement combinations can occur, out of the potentially very large number of possibilities that could result from unrestrained combinations of the activity of the 600 or so skeletal muscles we have (Bizzi et al., 1991). Therefore, such dumb "training" from muscle twitch combinations and the resulting co-occurring cortical activity can guide cortico-cortical connections and establish motor combinations that will be used later in life. Once the basic Bauplan is established, spindles become "internalized" and largely disengage from body inputs, but persist during early stages of sleep and may continue to perform a similar function (Khazipov et al. 2004). Because morphology of the body changes throughout the life span, changes in the periphery are coordinated with changes in the nervous system to maintain the match between them. Another example of early coordinated motor activity is non-nutritive sucking behavior, which emerges at 13 weeks in utero in humans. Its pattern-generating circuit is located in the brainstem, and similar to baby kicks, the oromotor bouts trigger thalamocortical oscillations (Grassi et al., 2016; Moore et al., 2014). Coordinated changes in motor control and peripheral structures are striking in animals such as insects or frogs, whose larval and adult bodies differ (Truman 1992; Stehouwer 1992). Patterns of motor neuronal outputs are transformed significantly by the properties of the body (Zajac 1993). A remarkable example is the transformation from quadrupedal to bipedal locomotion in dogs and bears with loss or disuse of leg(s) during early development.
Links to examples of transformation from quadrupedal to bipedal locomotion.
https://www.youtube.com/watch?v=2WLErtXO1wo
https://www.youtube.com/watch?v=1f8xSZNHVRo
https://www.youtube.com/watch?v=gXienXTA4M0
https://www.youtube.com/watch?v=kcIkQaLJ9r8

Note 2. Body part illusion experiments also support the primacy of action even in adults with a well-developed somatosensory "map". When participants perform a repetitive finger movement with their hidden index finger under the table while a yoked rubber hand is placed in full view displaying the corresponding finger movements, after a few trials they experience the moving rubber hand as their own ("ownership") and that they are directly controlling its movements voluntarily ("agency"; Kalckert & Ehrsson, 2012, 2014). When the experimenter visibly touches the rubber finger with a paintbrush and, at the same time and unknown to the participant, also touches her hidden hand with another paintbrush multiple times, the participant voluntarily accepts the rubber hand as her own (Botvinick and Cohen 1998). The "rubber hand feels as touched" even though no somatosensory signals arrive from it to the brain.

If a temporal delay is inserted between voluntary finger movement and the yoked finger movement of the rubber hand, the feeling of ownership and agency over the avatar hand does not emerge. Further, by manipulating the type of movement (active or passive) and spatial-anatomical orientation of the rubber hand with respect to the and real hand (congruent or incongruent) the sense of body ownership and agency can be manipulated separately (Kalckert and Ehrsson, 2014, 2017). Overall, these experiments demonstrate that utility and meaning of inputs to the brain arise via actions.

Note 3. *Avatar limb illusion*. A complementary condition to the alien hand illusion (in normal subjects) is the "alien limb" syndrome as depicted somewhat unrealistically in the film Dr. Strangelove (1968). These patients lack agency of action and feel no ownership of a moving body part. Simple or complex movements of a limb are present but the patients may not be aware of the actions, occasionally leading to the denial of ownership of the limb itself (Biran and Chatterjee, 2004). Importantly, their feeling of agency and ownership for other body parts remain intact. When movement of the alien limb is pointed out to them, they report it as unintentional or unnoticed, or occasionally in opposition to their intentions. "The hand feels foreign" or "has a will of its own". Brain pathology typically involves damage to the medial frontal cortex and accompanying damage to the corpus callosum due to infarction in the territory supplied by the anterior cerebral artery, or corticobasal degeneration (Di Pietro et al., 2021). In fMRI investigations, the reduced sense of agency and alien limb severity are associated with structural and functional changes in the pre-supplementary cortical area and its connectivity with the prefrontal network (Abdulkarim et al., 2022; Wolpe et al., 2020). This syndrome illustrates that normal reafferentation from the body and even completely intact sensory system are not sufficient for perceiving the body without the brain being in full action control. In the reverse direction, the ownership of a phantom limb and the painful feeling that may arise from "it" after its loss or amputation is a well-studied clinical problem. If sensation was solely dependent on external inputs, phantom limbs would not be felt mentally (Brecht 2017; Ramachandran et al., 1995).

Note 4. *Scalable connectome.* Repurposing neuronal circuits in brains with growing complexity is invariably linked with the integration of the circuit in question with the newly added brain volume. A fundamental rule of brain organization is that local computation takes place under the constraints of global computation. In addition, local computation can be broadcasted either globally or selectively to virtually any brain circuits (Buzsaki 2006). This accomplishment depends on laying down the necessary wiring and sufficient numbers of synaptic connections to allow local-global interactions.

For example, breathing is commonly viewed as a simple motor function, sustained autonomously by a kernel of neurons in preBötzinger complex in the ventrolateral medulla (Yackle et al., 2017). How breathing is generated and where in the body such command mechanisms exist has fascinated researchers for centuries. Perhaps the first person to point into the right direction was the French physiologist Pierre Flourens, who thought that respiration is controlled by the 'noeud vital' located in the medulla oblongata (Flourens, 1858). This hypothesis gained experimental support by observing that specific lesions affected breathing (Lumsden, 1923). Locating the pattern generator in the preBötzinger nucleus had to wait several decades.

Because breathing is performed by the coordinated action of approximately the same number of muscles in all mammals, from the mouse to the whale, one might expect that the same number of neurons could manage respiration, irrespective of body size. Yet, the volume of the preBötzinger complex increases proportionally with brain size. Why are more neurons needed in larger animals to control for the same type of rhythmic function? A reasonable answer is the bidirectional communication between the medulla respiratory neurons and the rest of the brain. The otherwise autonomous respiration can be controlled voluntarily, for example, during speech production or the playing of a wind instrument and affected by emotion (Arshamian et al. , 2018; Brown and Gerbarg, 2009; Johannknecht and Kayser, 2021; Nakamura et al., 2018; Nardi et al., 2009; Herrero et al., 2017; Perl et al., 2019; Zelano et al., 2016). In the reverse direction, breathing has long been known to influence higher order behaviors. Neurons in preBötzinger complex can entrain neurons in a large number of brain regions (Biskamp et al., 2017; Heck et al., 2019; Ito et al., 2014; Kleinfeld et al., 2014; Karalis and Sirota, 2022; Liu et al., 2017; Tort et al., 2018). This effect may be conveyed by corollary messages inside the brain rather than through exafferentation from the diaphragm or intercostal muscles (Huf et al., 2022). Supplying sufficient numbers and length of axons requires multiplication of these neurons. Conversely, numerous brain circuits in growing brains keep their access to respiratory neurons requiring an increase in both neuron size and number.

This reasoning likely applies to other neuron groups with “simple” basic functions, such as neuromodulators. The numbers of neurons in the locus coeruleus grow from 1,600 in the rat (unilaterally) to 50,000 in humans and are correlated with the size of the medulla and neocortical gray matter (Foote et al., 1983; Sharma et al., 2010). The number of neurons in VTA has increased from 25000 neurons in rats to 2.5 million in humans (Halliday and Törk 1986). This “access need” hypothesis can explain why spiking activity in virtually identical neurons can have so many different correlates, such as place fields and odor-specific responses in VTA dopamine neurons (Fujisawa et al., 2011).

An alternative solution to send longer axons and receive more inputs would be to increase neuron size. Examples include Betz cells of the primary motor cortex in large body size animals, where connecting the spinal cord segments at increased length require longer and thicker axons to increase axon conduction velocity and, thus, the ability of sending spikes within constant time frames independent of body size. However, increasing neuron size may have metabolic limitations (Fonseca-Azevedo et al., 2012). In contrast, increasing neuronal numbers, which can project to different targets and receive inputs from multiple targets by subsets of neurons come with circuit versatility and increased computation power. The increased number of projection neurons, linked by local axons and local interneurons allow for flexibly broadcasting local computation globally or to specific targets.

Note 5. A hungry newborn baby does not care about the beauty or complexity of the world. Its brain generates an action in the form of crying when a homeostatic need (an internal sense) is not being met. This simple action is sufficient because evolution has equipped the baby’s niche—the environment into which a baby is born—with a caregiver. Perceiving and recognizing the mother’s face and other close family members comes weeks later (Burnham, 1993).

Note 6. *Actions Dominate Waking Brain Activity*
In the somatosensory barrel cortex, intermingled populations of neurons respond not only to whisker touch but also to whisker movement, key components of object identification and navigation by active touch. The corollary template from the vibrissal motor cortex is projected to layer 1 of the barrel cortex (vS1; Petreanu et al., 2012). In auditory structures, one's own actions have long been known to affect sound-induced responses (Schneider and Mooney, 2015). In the visual cortex of mice, locomotion magnifies visual responses via changes in gain (Niell and Stryker, 2010), and activity of many neurons varies with movement speed (Saleem et al., 2013). Both cortical and subcortical activity is dominated by uninstructed movements not required for the experimenter-specified task. Trial-by-trial fluctuations to sensory inputs, often considered to be due to 'noise', can be accounted for by "spontaneous" movement variables, especially by paw movements, licking and facial muscle twitches as well as pupil diameter, reflecting the effect of the autonomic nervous system on sensory responses (Stringer et al., 2019).

Most of these studies have been performed in the head-fixed preparation, which conveniently allows large-scale recording but confine free movement. However, head movements are critical to adjust visual, auditory and odor sensors in an optimal position, particularly during navigation (Meyer et al., 2018). During head movements, layer 6 principal neurons in mouse primary visual cortex respond to the angular velocity of horizontal rotation. The motor command signal is conveyed to the visual cortex by way of the retrosplenial cortex and combined with visual information in a linear manner (Vélez-Fort et al., 2018). An upstream system of the retrospenial cortex is the entorhinal-hippocampal system, which relies heavily on motion signals (McNaughton et al., 1996). This visual-motion processing path is therefore a corollary discharge system, reporting self-generated inputs to higher order and sensory systems. In more complex brains, eye movements are the main source of visual exploration. During saccadic eye movements, transfer of visual information from the retina is suppressed, followed by a transient gain in circuit computation in visual and related areas (Leopold and Logothetis, 1998). Overall, these findings indicate that action commands profoundly shape neural activity throughout the brain and that corollary feedback from action systems to classical sensory areas is a more significant component than "exafferentation" conveyed by the sensors about the environment. Sensory cortices alone cannot veridically "represent" the body or the external world. Analogous to the highly distributed efference copy for muscle activation, there is likely also a widely distributed reafference for the organism's endocrine and immune states.

Note 7. Many different movement types affect wide territories of brain activity. Simple actions, such as optokinetic nystagmus have been shown to activate numerous of subcortical and cortical structures (Macé et al., 2018). The magnitude of responses to whisker touch in the barrel cortex varies extensively with the phase of the voluntary whisking cycle and activity in several other brain areas is modulated by whisking (Curtis and Kleinfeld 2009; Swed et al., 2003). Even in the absence of touch, the phase, deflection amplitude, and velocity of whisker protraction matched the landing distance in a gap jump task, likely coordinated with a central prediction (or error)

signal computed by the phase reset of hippocampal theta oscillation cycles (Voigst et al., 2015; Green et al., 2022).

Note 8. Movement-induced gain modulation is also present in the visual thalamus and superior colliculus (Erisken et al. 2014; Savier et al. 2019). Subsets of visual cortical neurons respond to combination of movement and location of the animal, resembling hippocampal place cells (Saleem et al., 2018). Visual inputs do not interrupt motor responses but, instead, sensory and motor responses fuse in orthogonal dimensions (Stringer et al., 2019).

Note 9. An extreme example of the role of locomotion on the hippocampal navigational system is the disappearance of place cell activity when animals are immobilized (McNaughton et al., 1996).

Abdulkarim Z, Guterstam A, Hayatou Z, Ehrsson HH. Neural substrates of body ownership and agency during voluntary movement. BioRrxiv July 23, 2022. https://doi.org/10.1101/2022.07.23.501221

Arshamian A, Iravani B, Majid A, Lundstrom JN (2018) Respiration Modulates olfactory memory consolidation in humans. J Neurosci 38:10286–10294.

Ayaz, A., Saleem, A.B., Schölvinck, M.L., Carandini, M., 2013. Locomotion controls spatial integration in mouse visual cortex. Curr. Biol. CB 23, 890–894.

Bastos, A.M., Usrey, W.M., Adams, R.A., Mangun, G.R., Fries, P., Friston, K.J., 2012. Canonical microcircuits for predictive coding. Neuron 76, 695–711.

Biran I, Chatterjee A. Alien hand syndrome. Archives of Neurology, 61 (2) (2004), p. 292.

Biskamp J, Bartos M, Sauer JF (2017) Organization of prefrontal network activity by respiration-related oscillations. Sci Rep 7:45508.

Bouvier, G., Senzai, Y., Scanziani, M., 2020. Head Movements Control the Activity of Primary Visual Cortex in a Luminance-Dependent Manner. Neuron 108, 500-511.

Brecht M (2017) The body model theory of somatosensory cortex. Neuron 94:985– 992.

Brown RP, Gerbarg PL. Yoga breathing, meditation, and longevity. Ann N Y Acad Sci. 2009;1172:54–62.

Burnham D. Visual recognition of mother by young infants: facilitation by speech. Perception. 1993;22(10):1133-53.

Cisek, P., 2019. Resynthesizing behavior through phylogenetic refinement. Atten. Percept. Psychophys. 81, 2265–2287.

Cisek, P., Kalaska, J.F., 2010. Neural mechanisms for interacting with a world full of action choices. Annu. Rev. Neurosci. 33, 269–298.

Di Pietro M, Russo M, Dono F, Carrarini C, Thomas A, Di Stefano V, Telese R, Bonanni L, Sensi SL, Onofrj M, Franciotti R. A Critical Review of Alien Limb-Related Phenomena and Implications for Functional Magnetic Resonance Imaging Studies. Front Neurol. 2021 Sep 9;12:661130.

Erisken S, Vaiceliunaite A, Jurjut O, Fiorini M, Katzner S, Busse L. Effects of locomotion extend throughout the mouse early visual system. Curr Biol. 2014 Dec 15;24(24):2899-907.

Flourens M. Nouveau details sur le noeud vital. Compt Rend Acad Sci. 1858;47:803–6.
Fonseca-Azevedo K, Herculano-Houzel S. Metabolic constraint imposes tradeoff between body size and number of brain neurons in human evolution. Proc Natl Acad Sci U S A. 2012 Nov 6;109(45):18571-6

Fujisawa S, Buzsáki G. A 4 Hz oscillation adaptively synchronizes prefrontal, VTA, and hippocampal activities. Neuron. 2011 Oct 6;72(1):153-65.

Grassi A, Cecchi F, Sgherri G, Guzzetta A, Gagliardi L, Laschi C. Sensorized pacifier to evaluate non-nutritive sucking in newborns. Med Eng Phys. 2016 Apr;38(4):398-402.

Halliday GM, Törk I. Comparative anatomy of the ventromedial mesencephalic tegmentum in the rat, cat, monkey and human. J Comp Neurol. 1986 Oct 22;252(4):423-45

Heck DH, Kozma R, Kay LM (2019) The rhythm of memory: how breathing shapes memory function. J Neurophysiol 122:563–571.

Hernandez-Peon, R., Scherrer, H., Jouvet, M., 1956. Modification of electric activity in cochlear nucleus during attention in unanesthetized cats. Science 123, 331–332.

Herrero JL, Khuvis S, Yeagle E, Cerf M, Mehta AD (2017) Breathing above the brainstem: volitional control and attentional modulation in humans. J Neurophysiol 119:145–159.

Huf A, Karlen-Amarante M, Pitts T, Ramirez JM (2022) Optogenetic stimulation of pre–Bötzinger complex reveals novel circuit interactions in swallowing–breathing coordination. Proc Natl Acad Sci U S A 119:e2121095119.

Ito J, Roy S, Liu Y, Cao Y, Fletcher M, Boughter JD Jr, Grun S, Heck DH (2014) Whisker barrel cortex delta oscillations and gamma power in the awake mouse are linked to respiration. Nat Commun 5:3572.

Johannknecht M, Kayser C (2021) The influence of the respiratory cycle on reaction times in sensory-cognitive paradigms. BioRxiv 2021.05.06.442800v1.

Kahana, M.J., 1996. Associative retrieval processes in free recall. Mem. Cognit. 24, 103–109.

Kalckert, A., & Ehrsson, H. H. (2014). The moving rubber hand illusion revisited: Comparing movements and visuotactile stimulation to induce illusory ownership. Consciousness and Cognition, 26, 117–132.

Kalckert, A., & Ehrsson, H. H. (2017). The Onset Time of the Ownership Sensation in the Moving Rubber Hand Illusion. Frontiers in Psychology, 8. https://doi.org/10.3389/fpsyg.2017.00344

Karalis N, Sirota A. Breathing coordinates cortico-hippocampal dynamics in mice during offline states. Nat Commun. 2022 Jan 24;13(1):467.
Katz LC, Shatz CJ. Synaptic activity and the construction of cortical circuits. Science. 1996 Nov 15;274(5290):1133-8.

Keller, G.B., Mrsic-Flogel, T.D., 2018. Predictive Processing: A Canonical Cortical Computation. Neuron 100, 424–435.

Khazipov R, Sirota A, Leinekugel X, Holmes GL, Ben-Ari Y, Buzsáki G. Early motor activity drives spindle bursts in the developing somatosensory cortex. Nature. 2004; 432(7018):758-61.

Killian, N.J., Potter, S.M., Buffalo, E.A., 2015. Saccade direction encoding in the primate entorhinal cortex during visual exploration. Proc. Natl. Acad. Sci. 112, 15743–15748.

Kleinfeld D, Deschênes M, Wang F, Moore JD. More than a rhythm of life: breathing as a binder of orofacial sensation. Nat Neurosci. 2014 May;17(5):647-51.

Leinweber, M., Ward, D.R., Sobczak, J.M., Attinger, A., Keller, G.B., 2017. A Sensorimotor Circuit in Mouse Cortex for Visual Flow Predictions. Neuron 95, 1420-1432.

Liu Y, McAfee SS, Heck DH (2017) Hippocampal sharp-wave ripples in awake mice are entrained by respiration. Sci Rep 7:8950.

Lumsden T. The regulation of respiration: Part I. J Physiol. 1923;58:81–91.

Martikainen, M.H., Kaneko, K., Hari, R., 2005. Suppressed Responses to Self-triggered Sounds in the Human Auditory Cortex. Cereb. Cortex 15, 299–302.

Milh M, Kaminska A, Huon C, Lapillonne A, Ben-Ari Y, Khazipov R. Rapid cortical oscillations and early motor activity in premature human neonate. Cereb Cortex. 2007 Jul;17(7):1582-94.

Moore, JD Kleinfeld D, Wang F. How the brainstem controls orofacial behaviors comprised of rhythmic actions. Trends Neurosci. **37**, 370–380 (2014).

Nakamura NH, Fukunaga M, Oku Y (2018) Respiratory modulation of cognitive performance during the retrieval process. PLoS ONE 13:e0204021.

Nardi E, Freire RC, Zin WA. Panic disorder and control of breathing. Respir Physiol Neurobiol. 2009;167:133–143.

Pakan, J.M.P., Currie, S.P., Fischer, L., Rochefort, N.L., 2018. The Impact of Visual Cues, Reward, and Motor Feedback on the Representation of Behaviorally Relevant Spatial Locations in Primary Visual Cortex. Cell Rep. 24, 2521–2528.

Perl O, Ravia A, Rubinson M, Eisen A, Soroka T, Mor N, Secundo L, Sobel N (2019) Human non-olfactory cognition phase-locked with inhalation. Nat Hum Behav 3(5): 501–512.

Ramachandran VS, Rogers- Ramachandran D, Cobb S (1995) Touching the phantom limb. Nature 377:489– 490.

Saleem AB, Diamanti EM, Fournier J, Harris KD, Carandini M. Coherent encoding of subjective spatial position in visual cortex and hippocampus. Nature. 2018 Oct;562(7725):124-127.

Saleem, A.B., Ayaz, A., Jeffery, K.J., Harris, K.D., Carandini, M., 2013. Integration of visual motion and locomotion in mouse visual cortex. Nat. Neurosci. 16, 1864–1869.

Savier EL et al. (2019) Effects of Locomotion on Visual Responses in the Mouse Superior Colliculus. *J. Neurosci* 39, 9360–936.

Snodderly, D.M., Kagan, I., Gur, M., 2001. Selective activation of visual cortex neurons by fixational eye movements: implications for neural coding. Vis. Neurosci. 18, 259–277.

Sommer, M.A., Wurtz, R.H., 2002. A pathway in primate brain for internal monitoring of movements. Science 296, 1480–1482.

Stringer C, Pachitariu M, Steinmetz N, Reddy CB, Carandini M, Harris KD. Spontaneous behaviors drive multidimensional, brainwide activity. Science. 2019 Apr 19;364(6437):255.

Stringer, C., Pachitariu, M., Steinmetz, N., Reddy, C.B., Carandini, M., Harris, K.D., 2019. Spontaneous behaviors drive multidimensional, brainwide activity. Science 364, 255.

Tort ABL, Brankack J, Draguhn A (2018) Respiration-entrained brain rhythms are global but often overlooked. Trends Neurosci 41:186–197.

Varga S, Heck DH (2017) Rhythms of the Body, Rhythms of the Brain: Respiration, Neural Oscillations, and Embodied Cognition. Conscious Cogn 56:77–90.

Vélez-Fort, M., Bracey, E.F., Keshavarzi, S., Rousseau, C.V., Cossell, L., Lenzi, S.C., Strom, M., Margrie, T.W., 2018. A Circuit for Integration of Head- and Visual-Motion Signals in Layer 6 of Mouse Primary Visual Cortex. Neuron 98, 179-191.

Vinck, M., Batista-Brito, R., Knoblich, U., Cardin, J.A., 2015. Arousal and locomotion make distinct contributions to cortical activity patterns and visual encoding. Neuron 86, 740–754.

Webb, B., 2004. Neural mechanisms for prediction: do insects have forward models? Trends Neurosci. 27, 278–282.

Weyand, T.G., Malpeli, J.G., 1993. Responses of neurons in primary visual cortex are modulated by eye position. J. Neurophysiol. 69, 2258–2260.

Wolpe N, Hezemans FH, Rowe JB. Alien limb syndrome: A Bayesian account of unwanted actions. Cortex. 2020 127:29-41.

Yackle K, Schwarz LA, Kam K, Sorokin JM, Huguenard JR, Feldman JL, Luo L, Krasnow MA. Breathing control center neurons that promote arousal in mice. Science 2017 Mar 31;355(6332):1411-1415

Zelano C, Jiang H, Zhou G, Arora N, Schuele S, Rosenow J, Gottfried JA (2016) Nasal respiration entrains human limbic oscillations and modulates cognitive function. J Neurosci 36:12448–12467.

COGNITION FROM ACTION

Note 1. Conceptualizing thinking as a body-disengaged operation (Buzsaki 2019), may explain why the target circuits of prefrontal cortical areas are so similar to those of the motor cortex with their dutiful corollary efferent connections to the rest of the brain (Passingham et al. 2002; Miller and Cohen, 2001). In the cognitive literature, these internal connections are referred to as "top-down" control (Miller and Cohen, 2001) but it is important to point out that they are analogous to the corollary discharge principle present even in the simplest brains (Fig. 1).

Note 2. Navigation in animals in which the hippocampus does not receive a corollary timing signal (e.g., echolocation in bats (Jones and Teeling, 2006) or saccadic eye movements in primates (Jutras et al., 2013) is invariably linked to hippocampal theta oscillations (an "internal timer"), the cycles of which contain sweeps of time (phase)-organized neural sequences corresponding to segments of the animal's travel along past, present, and future locations (Dragoi & Buzsáki 2006; O'Keefe & Recce 1993; Skaggs & McNaughton 1996).

Note 3. *Action-based classification of memory*. The value of a memory is only as good as its retrievability. While working-memory representations typically vanish immediately after being transformed into action, with no further service to the organism, other information enters long-term storage, and with support from the hippocampus, this knowledge can then assist with multiple future actions, including creating novel ideas. Thus, distinguishing memory mechanisms based on their single utility or multiple utilities is perhaps more meaningful than distinguishing between working and long-term memories (Buzsaki et al., 2022).

Note 4. Replay of waking experience during sleep corresponds to a correlation between rank order of spike sequences during the different brain states. These correlations are often considered to be acquired from learning experience. However, it has been noted that the correlations

between spike sequences across sleep episodes with interleaving waking are much higher than between learning and post-learning sleep (Hirase et al. 2001; Kudrimoti et al., 1999; Lee & Wilson, 2002; Luczak et al., 2007; Nadasdy et al., 1999; Wilson & McNaughton, 1994). Furthermore, wake-sleep correlations are higher in the neocortex (Euston et al., 2007; Ji and Wilson, 2007), striatum (Lansink et al., 2009; Pennartz et al., 2011; van der Meer et a., 2010), head directions system and entorhinal cortex (Gardner et al., 2019). The head-direction (HD) system functions as a compass, with member neurons robustly increasing their firing rates when the animal's head points in a specific direction (Ranck 1985). The reference of the compass (virtual 0 degree) is specific to each environment, determined by the constellation of sensory cues, and the activity packet varies systematically when the animal turns its head from side to side. However, this sequential firing is preserved during sleep, revealing its internally organized nature. During REM, the spontaneous drift of the activity packet is similar to that observed during waking but accelerates tenfold during nonREM sleep. From a population of head direction neurons one can decode the animal's actual head orientation, and infer a 'virtual gaze' during REM—that is, which direction the mouse is 'looking' (Peyrache et al., 2015). Indeed, the changes in the ensemble activity of head direction neurons are in register with the direction of eye movements both in the waking animal and REM sleep (Senzai & Scanziani, 2022). The invariance of the correlation structure of the head direction neurons across environments and brain states, independent of specific sensory inputs demonstrates an internally organized continuous attractor dynamic ("ring attractor"; Knierim and Zhang 2012; Peyrache et al., 2015).

The head direction sense is fundamental in navigation and also present in animals with small brains, such as insects. While in mammals, the ring attractor is virtual, in the fruit fly *Drosophila melanogaster* it is literally a ring, called central complex. The central complex is an unpaired, midline-spanning set of neuropils that consist of the protocerebral bridge, the ellipsoid body, the fan-shaped body and the paired noduli. The protocerebral bridge is organized in 16-18 contiguous glomeruli and the ellipsoid body in eight adjoined tiles. The central complex implicated in many navigation-related processes, and it has been shown that the activity 'bump' moves from one group of neurons to the next as the animal rotates with respect to its surrounding even when the fly walks in darkness (Seelig and Jayaraman, 2015; Giraldo et al., 2018).

The head direction system is a prime example how neuronal activity can be transduced by combining different types of computation. Investigators observed that some neurons in the entorhinal cortex fire specifically near walls ("border cells"; Solstad et al., 2008; Savelli et al., 2008) or predicted that there should be dedicated a neuron "type" that signals the boundaries of the environment ("boundary vector cells"; Lever et al., 2009). These are also head direction neurons, which are modulated by the affordances and constraints of the walls or gaps, where the animal can walk only parallel (and not across) the border and combine head direction with visual and haptic flow ("the wall is on my right"; Peyrache et al., 2017).

Note 5. Since head direction neurons are characterized by a 60°-wide correlated neuronal firing (activity packet) in all brain states, it was suggested that entorhinal grid cell organization also depends on head direction information (Peyrache et al., 2015). Indeed, lesion or inactivation of thalamic head direction neurons disrupted grid cell organization (Winter et al., 2015). Further support for the intricate dependence of entorhinal grid cells on the head direction organization comes from subsequent observations that the spatial phase offsets of grid cells in the waking

animal correlates strongly with spike rate correlations of these neurons during both REM and nonREM sleep with the same magnitude of nonREM compression than in the case of head direction neurons. Similarly, brain state-invariant correlations between conjunctive grid-head direction and pure head direction cells in the medial entorhinal cortex were predicted by their head direction tuning offsets during waking (Gardner et al., 2019). These findings were extended by showing that the brain state invariance of the correlation structure of grid cells is maintained across environments, thus independent of specific sensory inputs. The joint activity of grid cells from an individual entorhinal module resides on a toroidal manifold. Positions on the torus correspond to positions of the moving animal in the environment and are maintained between environments and from wakefulness to sleep. Thus, the one-dimensional ring attractor is transduced onto network dynamics on a toroidal manifold (Gardner et al., 2022).

Overall, these findings demonstrate the dominance of preconfigured dynamics, which can provide a flexible scaffold to which individual experiences can be matched. Learning thus does not require synthesizing neuronal sequences from scratch but only minor modifications of a preexisting backbone structure (Dragoi and Tonegawa, 2011; Grosmark and Buzsaki, 2016).

Bayne, T., Brainard, D., Byrne, R.W., Chittka, L., Clayton, N., Heyes, C., Mather, J., Ölveczky, B., Shadlen, M., Suddendorf, T., Webb, B., 2019. What is cognition? Curr. Biol. CB 29, R608–R615.

Buzsáki, G., 1989. Two-stage model of memory trace formation: a role for "noisy" brain states. Neuroscience 31, 551–570.

Buzsáki, G., 2005. Theta rhythm of navigation: link between path integration and landmark navigation, episodic and semantic memory. Hippocampus 15, 827–840.

Curtis JC and Kleinfeld D. (2009) Phase-to-rate transformations encode touch in cortical neurons of a scanning sensorimotor system. Nat. Neurosci 12, 492–501.

Dragoi G, Buzsáki G. Temporal encoding of place sequences by hippocampal cell assemblies. Neuron. 2006 Apr 6;50(1):145-57.

Dragoi G, Tonegawa S. Preplay of future place cell sequences by hippocampal cellular assemblies. Nature. 2011 Jan 20;469(7330):397-401

Gardner RJ, Hermansen E, Pachitariu M, Burak Y, Baas NA, Dunn BA, Moser MB, Moser EI. Toroidal topology of population activity in grid cells. Nature. 2022 Feb;602(7895):123-128.

Gardner RJ, Lu L, Wernle T, Moser MB, Moser EI. Correlation structure of grid cells is preserved during sleep. Nat Neurosci. 2019 Apr;22(4):598-608.

Giraldo YM, Leitch KJ, Ros IG, Warren TL, Weir PT, Dickinson MH. Sun Navigation Requires Compass Neurons in Drosophila. Curr Biol. 2018 Sep 10;28(17):2845-2852.

Grosmark AD, Buzsáki G. Diversity in neural firing dynamics supports both rigid and learned hippocampal sequences. Science. 2016 Mar 25;351(6280):1440-3.

Hirase H, Leinekugel X, Czurkó A, Csicsvari J, Buzsáki G. Firing rates of hippocampal neurons are preserved during subsequent sleep episodes and modified by novel awake experience. Proc Natl Acad Sci U S A. 2001 Jul 31;98(16):9386-90.

Jones G, Teeling EC. The evolution of echolocation in bats. Trends Ecol Evol. 2006 Mar;21(3):149-56.

Jutras MJ, Fries P, Buffalo EA. Oscillatory activity in the monkey hippocampus during visual exploration and memory formation. Proc Natl Acad Sci U S A. 2013 Aug 6;110(32):13144-9.

Knierim, J.J. & Zhang, K. Attractor dynamics of spatially correlated neural activity in the limbic system. Annu. Rev. Neurosci. **35**, 267–285 (2012).

Knierim, J.J., Kudrimoti, H.S. & McNaughton, B.L. Place cells, head direction cells and the learning of landmark stability. J. Neurosci. **15**, 1648–1659 (1995).

Kudrimoti HS, Barnes CA, McNaughton BL. Reactivation of hippocampal cell assemblies: effects of behavioral state, experience, and EEG dynamics. J Neurosci. 1999. 19(10):4090-101.

Lee AK, Wilson MA. Memory of sequential experience in the hippocampus during slow wave sleep. Neuron. 2002 Dec 19;36(6):1183-94.

Lever C, Burton S, Jeewajee A, O'Keefe J, Burgess N. Boundary vector cells in the subiculum of the hippocampal formation. *J. Neurosci.* 2009;29:9771–9777.

Luczak A, Barthó P, Marguet SL, Buzsáki G, Harris KD. Sequential structure of neocortical spontaneous activity in vivo. Proc Natl Acad Sci U S A. 2007 Jan 2;104(1):347-52.

Macé É, Montaldo G, Trenholm S, Cowan C, Brignall A, Urban A, Roska B. Whole-Brain Functional Ultrasound Imaging Reveals Brain Modules for Visuomotor Integration. Neuron. 2018 Dec 5;100(5):1241-1251.

Marois, R., Ivanoff, J., 2005. Capacity limits of information processing in the brain. Trends Cogn. Sci. 9, 296–305

Nádasdy Z, Hirase H, Czurkó A, Csicsvari J, Buzsáki G. Replay and time compression of recurring spike sequences in the hippocampus. J Neurosci. 1999 Nov 1;19(21):9497-507.

O'Keefe J, Recce ML. Phase relationship between hippocampal place units and the EEG theta rhythm. Hippocampus. 1993 Jul;3(3):317-30.

Peyrache A, Lacroix MM, Petersen PC, Buzsáki G. Internally organized mechanisms of the head direction sense. Nat Neurosci. 2015 Apr;18(4):569-75.

Peyrache A, Schieferstein N, Buzsáki G. Transformation of the head-direction signal into a spatial code. Nat Commun. 2017 Nov 24;8(1):1752.

Poulet, J.F.A., Hedwig, B., 2002. A corollary discharge maintains auditory sensitivity during sound production. Nature 418, 872–876.

Ranck, J.B. Head direction cells in the deep cell layer of dorsal presubiculum in freely moving rats. in Electrical Activity of Archicortex (eds. Buzsáki, G. & Vanderwolf, C.H.) 217–220 (Akademiai Kiado, 1985).

Redish, A.D., Elga, A.N. & Touretzky, D.S. A coupled attractor model of the rodent head direction system. Netw. Comput. Neural Syst. **7**, 671–685 (1996).

Savelli F, Yoganarasimha D, Knierim JJ. Influence of boundary removal on the spatial representations of the medial entorhinal cortex. Hippocampus. 2008;18:1270–1282.

Schneider, D.M., Mooney, R., 2015. Motor-related signals in the auditory system for listening and learning. Curr. Opin. Neurobiol. 33, 78–84.

Schneider, D.M., Mooney, R., 2018. How Movement Modulates Hearing. Annu. Rev. Neurosci. 41, 553–572.

Seelig, J., Jayaraman, V. Neural dynamics for landmark orientation and angular path integration. Nature **521,** 186–191 (2015).

Senzai Y and Scanziani M. A cognitive process occurring during sleep is revealed by rapid eye movements. Science 2022

Sharp, P.E., Blair, H.T. & Cho, J. The anatomical and computational basis of the rat head-direction cell signal. Trends Neurosci. **24**, 289–294 (2001).

Skaggs WE, Knierim JJ, Kudrimoti HS, McNaughton BL. A model of the neural basis of the rat's sense of direction. Adv Neural Inf Process Syst. 1995. 7:173-80.

Skaggs WE, McNaughton BL, Wilson MA, Barnes CA. Theta phase precession in hippocampal neuronal populations and the compression of temporal sequences. Hippocampus. 1996;6(2):149-72.

Solstad T, Boccara CN, Kropff E, Moser MB, Moser EI. Representation of geometric borders in the entorhinal cortex. Science. 2008. 322:1865–1868.

Stringer C, Pachitariu M, Steinmetz N, Reddy CB, Carandini M, Harris KD. Spontaneous behaviors drive multidimensional, brainwide activity. Science. 2019. 364(6437):255.

Szwed M et al. (2003) Encoding of vibrissal active touch. Neuron 40, 621–630.

Taube, J.S. Head direction cells recorded in the anterior thalamic nuclei of freely moving rats. J. Neurosci. **15**, 70–86 (1995).

Taube, J.S. The head direction signal: origins and sensory-motor integration. Annu. Rev. Neurosci. **30**, 181–207 (2007).

Taube, J.S., Muller, R.U. & Ranck, J.B. Jr. Head-direction cells recorded from the postsubiculum in freely moving rats. II. Effects of environmental manipulations. J. Neurosci. **10**, 436–447 (1990).

Wilson MA, McNaughton BL. Reactivation of hippocampal ensemble memories during sleep. Science. 1994 Jul 29;265(5172):676-9.

Winter SS, Clark BJ, Taube JS. Spatial navigation. Disruption of the head direction cell network impairs the parahippocampal grid cell signal. Science. 2015 Feb 20;347(6224):870-874.

Zhang K. Representation of spatial orientation by the intrinsic dynamics of the head-direction cell ensemble: a theory. J Neurosci. 1996 Mar 15;16(6):2112-26.

Zugaro, M.B., Arleo, A., Berthoz, A. & Wiener, S.I. Rapid spatial reorientation and head direction cells. J. Neurosci. **23**, 3478–3482 (2003).

Synchronization of Body-Brain States

Note 1. Hippocampal research has a rich history connecting the ambulatory features of the preparative state with hippocampal physiology (Vanderwolf, 1969). During such states, the neural computations of the hippocampus are organized by a ~8 Hz 'theta' oscillation, where the precise frequency of this computation is flexible, and moves in step with the rate of experience with the external world (Maurer et al., 2012).

Maurer, A.P., Burke, S.N., Lipa, P., Skaggs, W.E., Barnes, C.A., 2012. Greater running speeds result in altered hippocampal phase sequence dynamics. Hippocampus 22, 737–747.

Multiplexed Reader Mechanisms of Hippocampal Messages

Note 1. A long series of past works have examined the various potential roles of the lateral septum in effecting behavior. Following lateral septum lesion, (Brady & Nauta 1953)originally described behavioral consequences as 'general emotional reactivity' (see also; (Spiegel et al. 1940)). Subsequently different authors have attributed specific roles of the lateral septum in anxiety (Chee & Menard 2013; Parfitt et al. 2017), arousal (Li et al. 2015), aggression (Wong et al., 2016), contextual memory(Besnard et al. 2019; Jarrard 1993; Leutgeb & Mizumori 2002; Vouimba et al. 1998), food intake (Azevedo et al. 2020; Scopinho et al. 2008; Sweeney & Yang 2015, 2016; Terrill et al. 2016) spatial memory (Jaffard et al. 1996; Simon et al. 1986), sexual behavior (Tsukahara et al. 2014), sexually dimorphic social play (Veenema et al. 2013), social preference (Shin et al. 2018), social memory (Leroy et al. 2018; Lukas et al. 2013), reward and

addiction (Cornish & van den Buuse 1996; Heath 1963; Le Merrer et al. 2007; Luo et al. 2011; McGlinchey & Aston-Jones 2018; Olds & Milner 1954; Sartor & Aston-Jones 2012; Zahm et al. 2010), gastric motility (Gong et al. 2013), and endocrine responses to stress (Anthony et al. 2014; Usher et al. 1974; Yadin & Thomas 1996). How the lateral septum contributes to all these diverse behaviors remain to be addressed. One alternative is that multiple descending circuits operate in parallel, each with a unique behavioral function. Another, perhaps more likely, explanation is that only a single corticofugal computation is in place in the lateral septum and the outcome of its damage depends on the affordance of the circumstances, and contextually appropriate action selection (Luo et al. 2011).

Anthony TE, Dee N, Bernard A, Lerchner W, Heintz N, Anderson DJ. 2014. Control of stress-induced persistent anxiety by an extra-amygdala septohypothalamic circuit. *Cell*. 156(3):522–36

Azevedo EP, Tan B, Pomeranz LE, Ivan V, Fetcho R, et al. 2020. A limbic circuit selectively links active escape to food suppression. *eLife*. 9:e58894

Besnard A, Gao Y, TaeWoo Kim M, Twarkowski H, Reed AK, et al. 2019. Dorsolateral septum somatostatin interneurons gate mobility to calibrate context-specific behavioral fear responses. *Nat Neurosci*. 22(3):436–46

Brady JV, Nauta WJH. 1953. Subcortical mechanisms in emotional behavior: affective changes following septal forebrain lesions in the albino rat. *Journal of Comparative and Physiological Psychology*. 46:339–46

Chee S-SA, Menard JL. 2013. The histaminergic H1, H2, and H3 receptors of the lateral septum differentially mediate the anxiolytic-like effects of histamine on rats' defensive behaviors in the elevated plus maze and novelty-induced suppression of feeding paradigm. *Physiol Behav*. 116–117:66–74

Cornish JL, van den Buuse M. 1996. Regional expression of c-fos in rat brain following stimulation of the ventral tegmental area. *Neurosci Lett*. 220(1):17–20

Gong Y, Xu L, Wang H, Guo F, Sun X, Gao S. 2013. Involvements of the lateral hypothalamic area in gastric motility and its regulation by the lateral septum. *Gen Comp Endocrinol*. 194:275–85

Heath RG. 1963. Electrical self-stimulation of the brain in man. *The American Journal of Psychiatry*. 120:571–77

Jaffard R, Vouimba RM, Marighetto A, Garcia R. 1996. Long-term potentiation and long-term depression in the lateral septum in spatial working and reference memory. *Journal of* Physiology-Paris. 90(5):339–41

Jarrard LE. 1993. On the role of the hippocampus in learning and memory in the rat. Behavioral and Neural Biology. 60(1):9–26

Le Merrer J, Gavello-Baudy S, Galey D, Cazala P. 2007. Morphine self-administration into the lateral septum depends on dopaminergic mechanisms: Evidence from pharmacology and Fos neuroimaging. Behavioural Brain Research. 180(2):203–17

Leroy F, Park J, Asok A, Brann DH, Meira T, et al. 2018. A circuit from hippocampal CA2 to lateral septum disinhibits social aggression. *Nature*. 564(7735):213–18

Leutgeb S, Mizumori SJY. 2002. Context-specific spatial representations by lateral septal cells. *Neuroscience*. 112(3):655–63

Li W, Motelow JE, Zhan Q, Hu Y-C, Kim R, et al. 2015. Cortical network switching: possible role of the lateral septum and cholinergic arousal. *Brain Stimul*. 8(1):36–41

Lukas M, Toth I, Veenema AH, Neumann ID. 2013. Oxytocin mediates rodent social memory within the lateral septum and the medial amygdala depending on the relevance of the social stimulus: male juvenile versus female adult conspecifics. *Psychoneuroendocrinology*. 38(6):916–26

Luo AH, Tahsili-Fahadan P, Wise RA, Lupica CR, Aston-Jones G. 2011. Linking Context with Reward: A Functional Circuit from Hippocampal CA3 to Ventral Tegmental Area. *Science*. 333(6040):353–57

McGlinchey EM, Aston-Jones G. 2018. Dorsal Hippocampus Drives Context-Induced Cocaine Seeking via Inputs to Lateral Septum. *Neuropsychopharmacology*. 43(5):987–1000

Olds J, Milner P. 1954. Positive reinforcement produced by electrical stimulation of septal area and other regions of rat brain. *J Comp Physiol Psychol*. 47(6):419–27

Parfitt GM, Nguyen R, Bang JY, Aqrabawi AJ, Tran MM, et al. 2017. Bidirectional Control of Anxiety-Related Behaviors in Mice: Role of Inputs Arising from the Ventral Hippocampus to the Lateral Septum and Medial Prefrontal Cortex. *Neuropsychopharmacology*. 42(8):1715–28

Sartor GC, Aston-Jones GS. 2012. A septal-hypothalamic pathway drives orexin neurons, which is necessary for conditioned cocaine preference. *J Neurosci*. 32(13):4623–31

Scopinho AA, Resstel LBM, Corrêa FMA. 2008. α1-Adrenoceptors in the lateral septal area modulate food intake behaviour in rats. *British Journal of Pharmacology*. 155(5):752–56

Shin S, Pribiag H, Lilascharoen V, Knowland D, Wang X-Y, Lim BK. 2018. Drd3 Signaling in the Lateral Septum Mediates Early Life Stress-Induced Social Dysfunction. *Neuron*. 97(1):195-208.e6

Simon H, Taghzouti K, Le Moal M. 1986. Deficits in spatial-memory tasks following lesions of septal dopaminergic terminals in the rat. *Behavioural Brain Research*. 19(1):7–16

Spiegel EA, Miller HR, Oppenheimer MJ. 1940. Forebrain and rage reactions. *Journal of Neurophysiology*. 3(6):538–48

Sweeney P, Yang Y. 2015. An excitatory ventral hippocampus to lateral septum circuit that suppresses feeding. *Nat Commun*. 6:10188

Sweeney P, Yang Y. 2016. An Inhibitory Septum to Lateral Hypothalamus Circuit That Suppresses Feeding. *J Neurosci*. 36(44):11185–95

Terrill SJ, Jackson CM, Greene HE, Lilly N, Maske CB, et al. 2016. Role of lateral septum glucagon-like peptide 1 receptors in food intake. *Am J Physiol Regul Integr Comp Physiol*. 311(1):R124-132

Tsukahara S, Kanaya M, Yamanouchi K. 2014. Neuroanatomy and sex differences of the lordosis-inhibiting system in the lateral septum. *Frontiers in Neuroscience*. 8:

Usher DR, Lieblich I, Siegel RA. 1974. Pituitary-Adrenal Function after Small and Large Lesions in the Lateral Septal Area in Food-Deprived Rats. *NEN*. 16(3–4):156–64

Veenema AH, Bredewold R, De Vries GJ. 2013. Sex-specific modulation of juvenile social play by vasopressin. *Psychoneuroendocrinology*. 38(11):10.1016/j.psyneuen.2013.06.002

Vouimba RM, Garcia R, Jaffard R. 1998. Opposite effects of lateral septal LTP and lateral septal lesions on contextual fear conditioning in mice. *Behavioral Neuroscience*. 112:875–84

Yadin E, Thomas E. 1996. Stimulation of the lateral septum attenuates immobilization-induced stress ulcers. *Physiol Behav*. 59(4–5):883–86

Zahm DS, Becker ML, Freiman AJ, Strauch S, Degarmo B, et al. 2010. Fos after single and repeated self-administration of cocaine and saline in the rat: emphasis on the Basal forebrain and recalibration of expression. *Neuropsychopharmacology*. 35(2):445–63

Hippocampal SPW-Rs reduce peripheral glucose levels

Note 1. The first report on the potential impact of brain activity on peripheral glucose levels was published over a century ago. Kersten (1921) noted in human patients with epilepsy that petit mal episodes were followed by a drop in glucose levels (Suppl. Fig. 2), while a precipitous rise in blood glucose levels followed other seizures. It was not until 60 years later that direct electrical stimulation of the dorsal hippocampus in rabbits was shown to decrease glucose levels by 32-59% and increase insulin levels by 154-229% in the bloodstream, the effect size increasing in the hyperglycemic state (Seto et al. 1983). Another centennial anniversary is the first administration of insulin to a human on January 12, 2022 (Banting 1922).

Note 2. Regular oscillations in circulating glucose levels on the time scale of minutes can predict and influence physiological states. Current glucose level provides information about available energy for the brain for timely allostatic responses (Tingley et al., 2021).

Note 3. The brain works cooperatively with pancreatic islets to adjust glucose production, storage, and utilization. Data obtained from type 2 diabetes patients implicates both brain and islet dysfunction in this process. In addition to the vagal nerve, pancreatic islets are also innervated by the dorsal root ganglion sensory neurons of the spinal cord (Mirzadeh et al. 2022). Traditional treatments of type 2 diabetes are based on a pancreatic islet-focused view of glucose metabolism but recent works show that the brain can also modulate insulin-independent glucose disposal. Sympathetic nervous system output to the pancreatic beta cells suppresses both glucose-stimulated and basal insulin secretion, while parasympathetic output increases glucose-stimulated insulin secretion (Ahrén 2000; Chien et al., 2019). In addition to the pancreas, the vagal nerve extensively innervates the gastrointestinal tract and the liver as well (de Lartigue G. 2016; Lin et al., 2021; Shimazu 1986). Distinct sensory neurons of the gastrointenstinal tract respond to different gut-derived signals and that their activation contributes to the regulation of feeding and glucose metabolism. Opto- or chemogenetic stimulation of upper-gut-innervating glucagon-like peptide 1 receptor (GLP1R)-expressing vagal afferents, which relay anorexigenic signals to parabrachial nucleus neurons in the brainstem, reduced feeding. Conversely, their inhibition elevated blood glucose levels independent of food intake. In contrast, chemogenetically stimulating vagal afferents that synaptically engage tyrosine-hydroxylase-expressing neurons in the nucleus of the solitary tract (NTS) increased feeding (Chen et al., 2020; Dranse et al., 2018; Clemmensen et al., 2017; Kim et al., 2018; Soty et al., 2017).

Note 4. The mechanisms supporting glucose homeostasis are overlapping with thermoregulation and sleep, and cold also suppresses insulin secretion (Young et al., 1979). To maintain core body temperature, thermogenic tissues rapidly increase glucose utilization by brown adipose tissue and shivering by skeletal muscle. Enhanced glucose utilization is facilitated in part by increased insulin sensitivity in these tissues (Vallerand et al., 1987), needed to preserve core temperature in the cold (Maickel et al., 1967). Most of these research observations have been performed in waking animals but it is not well understood how these processes apply to sleep, and especially to REM sleep when brain temperature is rapidly elevated in the absence of muscle-produced heat (Petersen et al., 2022).

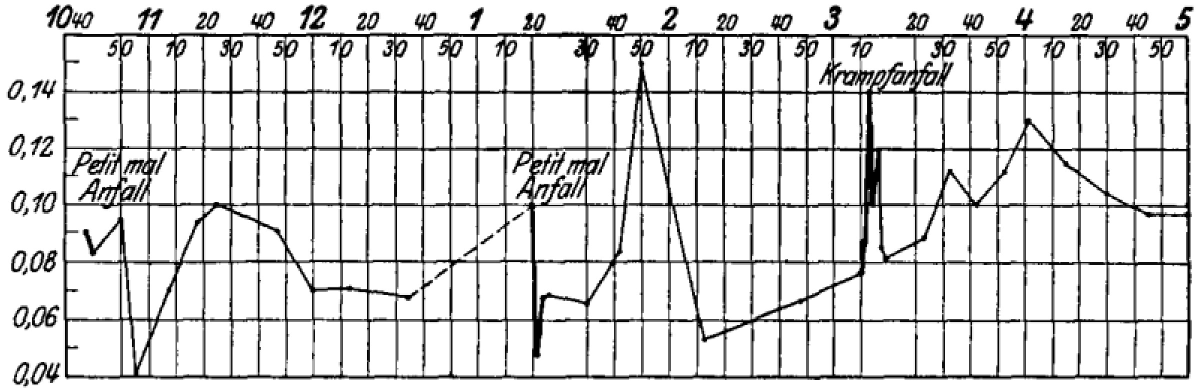

Abb. 3.

Supplementary Fig. 2. Peripheral blood sugar changes during petit mal seizures (*Anfall*) and generalized grand mal seizure (*Krampfanfall*) in an epileptic patient. Note large decreases of sugar levels after petit mal attacks. (Reproduced from Kersten 1921).

Ahrén B. 2000. Autonomic regulation of islet hormone secretion—implications for health and disease. Diabetologia 43(4):393–410

Banting FG, Best CH,Collip JB, Campbell WR, Fletcher AA. 1922. Pancreatic extracts in the treatment of diabetes mellitus. Can. Med. Assoc. J. 12(3):141–46.

Barrett LF, Simmons WK. Interoceptive predictions in the brain. Nat Rev Neurosci. 2015 Jul;16(7):419-29.

Bentsen, M. A., Mirzadeh, Z. & Schwartz, M. W. Revisiting How the Brain Senses Glucose-And Why. Cell Metab. 29, 11–17 (2019).

Bentsen, M.A., Rausch, D.M., Mirzadeh, Z., Muta, K., Scarlett, J.M., Brown, J.M., Herranz-Pérez, V., Baquero, A.F., Thompson, J., Alonge, K.M., Faber, C.L., Kaiyala, K.J., Bennett, C., Pyke, C., Ratner, C., Egerod, K.L., Holst, B., Meek, T.H., Kutlu, B., Zhang, Y., Sparso, T., Grove, K.L., Morton, G.J., Kornum, B.R., García-Verdugo, J.-M., Secher, A., Jorgensen, R., Schwartz, M.W., Pers, T.H., 2020. Transcriptomic analysis links diverse hypothalamic cell types to fibroblast growth factor 1-induced sustained diabetes remission. Nat. Commun. 11, 4458.

Berridge KC. Food reward: Brain substrates of wanting and liking. Neurosci Biobehav Rev Elsevier Ltd. 1996;20:1–25.

Boyd, K.A., O'Donovan, D.G., Doran, S., Wishart, J., Chapman, I.M., Horowitz, M., and Feinle, C. (2003). High-fat diet effects on gut motility, hormone, and appetite responses to duodenal lipid in healthy men. Am. J. Physiol. Gastrointest. Liver Physiol. 284, G188–G196.

Chen WG, Schloesser D, Arensdorf AM, Simmons JM, Cui C, Valentino R, Gnadt JW, Nielsen L, Hillaire-Clarke CS, Spruance V, Horowitz TS, Vallejo YF, Langevin HM. The Emerging

Science of Interoception: Sensing, Integrating, Interpreting, and Regulating Signals within the Self. Trends Neurosci. 2021. 44:3-16.

Chen, J., Cheng, M., Wang, L., Zhang, L., Xu, D., Cao, P., Wang, F., Herzog, H., Song, S., and Zhan, C. (2020). A vagal-NTS neural pathway that stimulates feeding. Curr. Biol. 30, 3986–3998.

Cheung, G.W., Kokorovic, A., Lam, C.K., Chari, M., and Lam, T.K. (2009). Intestinal cholecystokinin controls glucose production through a neuronal network. Cell Metab 10, 99–109.

Chien H-J, Chiang T-C, Peng S-J, Chung M-H, Chou Y-H, et al. 2019. Human pancreatic afferent and efferent nerves: mapping and 3-D illustration of exocrine, endocrine, and adipose innervation. Am. J. Physiol. Gastrointest. Liver Physiol. 317: G694–706

Clark KB, Naritoku DK, Smith DC, Browning RA, Jensen RA. Enhanced recognition memory following vagus nerve stimulation in human subjects. Nat Neurosci. 1999 Jan;2(1):94-8.

Clemmensen, C., M€uller, T.D., Woods, S.C., Berthoud, H.R., Seeley, R.J., and Tscho¨ p, M.H. (2017). Gut-brain cross-talk in metabolic control. Cell 168, 758–774.

Covasa, M. (2010). Deficits in gastrointestinal responses controlling food intake and body weight. Am. J. Physiol. Regul. Integr. Comp. Physiol. 299, R1423–R1439.

Davidson TL, Jones S, Roy M, Stevenson RJ. The cognitive control of eating and body weight: It’s more than what you think. Front Psychol. 2019;10.

de Lartigue G. 2016. Role of the vagus nerve in the development and treatment of diet-induced obesity. J. Physiol. 594(20):5791–815.

Dranse, H.J., Waise, T.M.Z., Hamr, S.C., Bauer, P.V., Abraham, M.A., Rasmussen, B.A., and Lam, T.K.T. (2018). Physiological and therapeutic regulation of glucose homeostasis by upper small intestinal PepT1-mediated protein sensing. Nat. Commun. 9, 1118.

Euston DR, Tatsuno M, McNaughton BL. Fast-forward playback of recent memory sequences in prefrontal cortex during sleep. Science. 2007 Nov 16;318(5853):1147-50.

Farabi, S. S., Carley, D. W. & Quinn, L. EEG power and glucose fluctuations are coupled during sleep in young adults with type 1 diabetes. Clin. Neurophysiol. Off. J. Int. Fed. Clin. Neurophysiol. 127, 2739–2746 (2016).

Girardeau G, Inema I, Buzsáki G. Reactivations of emotional memory in the hippocampus-amygdala system during sleep. Nat Neurosci. 2017 Nov;20(11):1634-1642.

Gomperts SN, Kloosterman F, Wilson MA. VTA neurons coordinate with the hippocampal reactivation of spatial experience. Elife. 2015 Oct 14;4:e05360.

Hall, J.L., Gonder-Frederick, L.A., Chewning, W.W., Silveira, J., Gold, P.E., 1989. Glucose enhancement of performance on memory tests in young and aged humans. Neuropsychologia 27, 1129–1138.

Heni M, Kullmann S, Ketterer C, Guthoff M, Linder K, Wagner R, Stingl KT, Veit R, Staiger H, Häring HU, Preissl H, Fritsche A. Nasal insulin changes peripheral insulin sensitivity simultaneously with altered activity in homeostatic and reward-related human brain regions. Diabetologia. 2012 Jun;55(6):1773-82

Ji D, Wilson MA. Coordinated memory replay in the visual cortex and hippocampus during sleep. Nat Neurosci. 2007 Jan;10(1):100-7.

Kersten H (1921). Ergebnisse zur Frage des elementaren Krampfes. Anstaltsarzt am Kinderheim Lewenberg (Schwerin).

Kim, K.S., Seeley, R.J., and Sandoval, D.A. (2018). Signalling from the periphery to the brain that regulates energy homeostasis. Nat. Rev. Neurosci. 19, 185–196.
Kishi, T. et al. Topographical organization of projections from the subiculum to the hypothalamus in the rat. J. Comp. Neurol. 419, 205–222 (2000).

Knigge, K.M., 1961. Adrenocortical response to stress in rats with lesions in hippocampus and amygdala. Proc. Soc. Exp. Biol. Med. Soc. Exp. Biol. Med. N. Y. N 108, 18–21.

Kullmann, S. et al. Selective insulin resistance in homeostatic and cognitive control brain areas in overweight and obese adults. Diabetes Care 38, 1044–1050 (2015).

Lansink CS, Goltstein PM, Lankelma JV, McNaughton BL, Pennartz CM. Hippocampus leads ventral striatum in replay of place-reward information. PLoS Biol. 2009. 7(8):e1000173.

Liebling, D.S., Eisner, J.D., Gibbs, J., and Smith, G.P. (1975). Intestinal satiety in rats. J. Comp. Physiol. Psychol. 89, 955–965.

Lin EE, Scott-Solomon E, Kuruvilla R. 2021. Peripheral innervation in the regulation of glucose homeostasis. Trends Neurosci. 44(3):189–202

Luczak A, Barthó P, Harris KD. Spontaneous events outline the realm of possible sensory responses in neocortical populations. Neuron. 2009 May 14;62(3):413-25.

Luczak A, Barthó P, Marguet SL, Buzsáki G, Harris KD. Sequential structure of neocortical spontaneous activity in vivo. Proc Natl Acad Sci U S A. 2007 Jan 2;104(1):347-52.

Maickel RP,MatussekN, Stern DN,Brodie BB. 1967. The sympathetic nervous system as a homeostatic mechanism. I. Absolute need for sympathetic nervous function in body temperature maintenance of cold-exposed rats. J. Pharmacol. Exp. Ther. 157(1):103–10.

McCorry, L.K., 2007. Physiology of the Autonomic Nervous System. Am. J. Pharm. Educ. 71, 78.

McFarland, J.M., Bondy, A.G., Saunders, R.C., Cumming, B.G., Butts, D.A., 2015. Saccadic modulation of stimulus processing in primary visual cortex. Nat. Commun. 6, 8110.

McIntire LK, McKinley RA, Goodyear C, McIntire JP, Brown RD. Cervical transcutaneous vagal nerve stimulation (ctVNS) improves human cognitive performance under sleep deprivation stress. Commun Biol. 2021 Jun 10;4(1):634.

Muller PA, Schneeberger M, Matheis F, Wang P, Kerner Z, Ilanges A, Pellegrino K, Del Mármol J, Castro TBR, Furuichi M, Perkins M, Han W, Rao A, Pickard AJ, Cross JR, Honda K, de Araujo I, Mucida D. Microbiota modulate sympathetic neurons via a gut-brain circuit. Nature. 2020 Jul;583(7816):441-446.

Parent MB, Higgs S, Cheke LG, Kanoski SE. Memory and eating: A bidirectional relationship implicated in obesity. Neurosci Biobehav Rev. 2022. 132:110-129.
Pennartz CM, Ito R, Verschure PF, Battaglia FP, Robbins TW. The hippocampal-striatal axis in learning, prediction and goal-directed behavior. Trends Neurosci. 2011. 34(10):548-59.

Petersen PC, Vöröslakos M, Buzsáki G. Brain temperature affects quantitative features of hippocampal sharp wave ripples. J Neurophysiol. 2022. 127(5):1417-1425.

Quigley KS, Kanoski S, Grill WM, Barrett LF, Tsakiris M. Functions of Interoception: From Energy Regulation to Experience of the Self. Trends Neurosci. 2021. 44(1):29-38.

Radley, J. J. & Sawchenko, P. E. A common substrate for prefrontal and hippocampal inhibition of the neuroendocrine stress response. J. Neurosci. 2001. 31, 9683–96.

Reimer, J., Froudarakis, E., Cadwell, C.R., Yatsenko, D., Denfield, G.H., Tolias, A.S., 2014. Pupil fluctuations track fast switching of cortical states during quiet wakefulness. Neuron 84, 355–362.

Saito, H., Kaba, H., Sato, T., Nojima, K., Li, C.-S., Seto, K., Kimura, F., Kawakami, M., 1990. Influence of Dorsal Hippocampal Stimulation and Dorsal Fornix Lesions on Hepatic Glucose Metabolism in Rabbits. Exp. Clin. Endocrinol. Diabetes 96, 113–116.

Sakaki, M., Yoo, H.J., Nga, L., Lee, T.-H., Thayer, J.F., Mather, M., 2016. Heart rate variability is associated with amygdala functional connectivity with MPFC across younger and older adults. NeuroImage 139, 44–52.

Scheen AJ, Byrne MM, Plat L, Leproult R, Van Cauter E. Relationships between sleep quality and glucose regulation in normal humans. Amer J Physiol. 1996; **271**(2):E261–E270.

Schwartz, M.W., Woods, S.C., Porte, D., Jr., Seeley, R.J., and Baskin, D.G. (2000). Central nervous system control of food intake. Nature 404: 661–671.

Sjulson L, Peyrache A, Cumpelik A, Cassataro D, Buzsáki G. Cocaine Place Conditioning Strengthens Location-Specific Hippocampal Coupling to the Nucleus Accumbens. Neuron. 2018 Jun 6;98(5):926-934.

Soty, M., Gautier-Stein, A., Rajas, F., and Mithieux, G. (2017). Gut-brain glucose signaling in energy homeostasis. Cell Metab 25, 1231–1242.

Tingley D, Peyrache A: On the methods for reactivation and replay analysis. Philos Trans R Soc B Biol Sci 2019 http://dx.doi. org/10.1098/rstb.2019.0231.

Vallerand AL, Perusse F, Bukowiecki LJ. 1987. Cold exposure potentiates the effect of insulin on in vivo glucose uptake.Am. J. Physiol. 253(2):E179–86

van der Meer MA, Kalenscher T, Lansink CS, Pennartz CM, Berke JD, Redish AD. Integrating early results on ventral striatal gamma oscillations in the rat. Front Neurosci. 2010 Sep 15;4:300.
Wang, P.Y., Caspi, L., Lam, C.K., Chari, M., Li, X., Light, P.E., Gutierrez-Juarez, R., Ang, M., Schwartz, G.J., and Lam, T.K. (2008). Upper intestinal lipids trigger a gut-brain-liver axis to regulate glucose production. Nature 452, 1012–1016.

Welch, I.M., Sepple, C.P., and Read, N.W. (1988). Comparisons of the effects on satiety and eating behavior of infusion of lipid into the different regions of the small intestine. Gut 29, 306–311.

Young JB, Landsberg L. 1979. Effect of diet and cold exposure on norepinephrine turnover in pancreas and liver.Am. J. Physiol. 236(5):E524–33

Zuniga A, Stevenson RJ, Thayer ZC, Miller LA, Francis HM, Saluja S, et al. The impact of hippocampal damage on appetitive control. Neurocase. 2020;26:305–12.

ROLE OF THE HIPPOCAMPUS IN BODY-BRAIN-BODY REGULATORY LOOPS

Hippocampus Modulates Body States

Britton, S.W., 1925. Studies on the conditions of activity in endocrine glands: XVII. The nervous control of insulin secretion. Am. J. Physiol. 291–308.

Corkill, A.B., Marks, H.P., White, W.E., 1933. Relation of the pituitary gland to the action of insulin and adrenaline 612, 193–205.

Güemes, A., Georgiou, P., 2018. Review of the role of the nervous system in glucose homoeostasis and future perspectives towards the management of diabetes. Bioelectron. Med. 4, 1–18.

Kim, S., Park, M.-J., 2017. Effects of growth hormone on glucose metabolism and insulin resistance in human. Ann. Pediatr. Endocrinol. Metab. 1012, 145–152.
Kuo, T., McQueen, A., Chen, T.-C., Wang, J.-C., 2015. Regulation of Glucose Homeostasis by Glucocorticoids. Adv Exp Med Biol 872, 99–126. https://doi.org/10.1007/978-1-4939-2895-8.

Langley, J.N., 1903. The Autonomic Nervous System. Brain 26, 1–26.
McNay, E.C., Gold, P.E., 2002. Food for thought: fluctuations in brain extracellular glucose provide insight into the mechanisms of memory modulation. Behav. Cogn. Neurosci. Rev. 1, 264–280.

Myers, M.G., Affinati, A.H., Richardson, N., Schwartz, M.W., 2021. Central nervous system regulation of organismal energy and glucose homeostasis. Nat. Metab. 3, 737–750.

Porter, R., 1954. The central nervous system and stress-induced eosinopenia. Recent Prog. Horm. Res. 10, 1–27.

Somers, V.K., Dyken, M.E., Mark, A.L., Abboud, F.M., 1993. Sympathetic-nerve activity during sleep in normal subjects. N. Engl. J. Med. 328, 303–307.

Hippocampus as an Internal Sensor of Body State

Note 1. The neuronal signaling often repurposes various molecules of the body. For example, the gut peptide cholecystokinin (CCK) possesses multiple functions in the gastrointestinal tract, including gallbladder contraction and countering insulin-induced hunger. In the brain, it is present in a subclass of inhibitory interneurons, and CCK inhibits expression of orexigenic peptides in the hypothalamus (Chandra & Liddle, 2007). Peripherally, the vasoactive intestinal peptide (VIP) functions as a vasodilator and also stimulates electrolyte secretion in the jejunum (Lu et al., 2022). In the hypothatalamic suprachiasmatic nucleus, VIP synchronizes neurons of the circadian clock via activating the cAMP–MAPK–CREB cascade (Antle, M. C. & Silver, 2005; Vosko et al., 2007) and is also an important signaling substance in another set of interneurons (Freund & Buzsaki, 1996). NPY, a neuropeptide, also potently stimulates feeding (Andermann and Lowell, 2017). In mammals, corticosteroids excreted by the adrenal glands are essential in maintaining the structural integrity of hippocampal granule cells (Sloviter et al., 1989). These and other examples illustrate the deep interdependence between brain and body. Even though brain architecture becomes modified in different ways in ever more complex brains, it continues to interact with the body's fundamental functions.

Note 2. Another example of exaptation is the relationship between physical pain and pain from social exclusion because several chemical pathways activated by physical pain and pain from social exclusion overlap, illustrating that the physical pain system may have been co-opted to motivate social animals to respond to threats to their inclusion in the group (MacDonald & Leary, 2005).

Note 3. The terms automatic and autonomous refer to an independent operation of the periphery from the brain's conscious control. Yet, certain autopilot functions, such as respiration, can be

brought under voluntary control, for example, during speech production. Nearly all other autonomic functions are affected by emotions, and, in turn, become a conscious experience (LeDoux 1995), either by enhanced feedback from the body or by the corollary reafference signaling path in the brain. When disengaged from direct effect on the body, these reafference mechanisms may be responsible for memory-induced emotional re-experience, including post-traumatic stress disorder (PTSD; Sherin & Nemeroff, 2011). While normal waking from sleep is preceded by a massive release of adrenocorticotropin and cortisol, similar to anticipation of stressful events, such anticipation can be brought under volitional control. In people who can accurately time the end of their night's sleep at will, without using an alarm clock, the concentration of adrenocorticotropin in the blood also surges before waking (Born et al., 1999). These findings illustrate not only conscious voluntary control over hormonal release mechanisms but also that such, presumably, cortical supervision continues throughout sleep. In summary, while the brain can disconnect its communication with the environment, its bidirectional communication with the body persists during sleep. Subconscious cortical control over autonomic, endocrine and immune systems is ubiquitous, and may be the mechanism underlying numerous placebo effects (Hróbjartsson & Gøtzsche, 2001).

Note 4. Hundreds of millions of neurons of a distributed "brain" control the alimentary system and gather inputs from the various chemical and mobility signals from the gut and regulate a plethora of functions, including food intake and energy homeostasis. This peripheral "gut system" is traditionally dealt with as separate from the brain despite the rapidly accumulating evidence for gut-brain-gut interactions (Alhadeff et al., 2021). Thus, the metaphor of "gut feeling" may indeed have a physiological basis. Similar to the hippocampus, cerebellum and basal ganglia (main Fig. 1), the peripheral nervous system can be conceived as a side loop to the "core" organization of the brain.

Note 5. The sleep-wake dichotomy is strongly related to other brain operations such as sympathetic-parasympathetic functions, arousal, somatic and autonomic motor control. A recent suggestion is "that a primary function of sleep is to suppress motor activity" (Liu and Dan, 2019). However, it has been reported that stereotypic wheel running is associated with a substantial reduction in firing rates among a large subpopulation of cortical neurons, associated with sleep-like delta power, especially at high speeds (Fisher et al., 2016; Lyamin et al., 2004). Higher functions areas thus can disengage while the rest of the brain works on 'autopilot'.

Note 6. The potential role of the hypothalamus in sleep and wake was first identified in patients affected by *encephalitis lethargica*, associated with insomnia, since the hypothalamic areas often degenerated in these patients (von Economo, 1930). In rats and cats, surgical lesion of the hypothalamic preoptic area (POA) also induced sleeplessness and it was named as the "sleep center" of the brain (Nauta, 1946; McGinty and Sterman, 1968). Its sleep-inducing effect is under the inhibitory control ventrolateral part of the posterior hypothalamus (Sallanon et al., 1989) and many other descending afferents, suggesting that a complex circuit is involved in the regulation of wake-sleep state changes. Sleep neurons in POA are believed to promote sleep by suppressing postulated arousal circuits, such as the histaminergic tuberomammillary neurons, serotoninergic neuron in raphe nucleus, and noradrenergic locus coeruleus neurons (Saper and Fuller, 2017). More contemporary experiments identified galanin-expressing neurons in POA as the key subset, since their chemogenetic stimulation promoted sleep (Kroeger et al., 2018). In

contrast, optogenetic stimulation of galanin neurons either promoted nonREM sleep (at < 4 Hz stimulation; Kroeger et al., 2018) or wakefulness (> 8 Hz; Chung et al., 2017), suggesting that even this cell "type" is mixed. Optogenetic stimulation of several other types of GABAergic neurons in POA (which also express cholecystokinin, CCK, corticotropin-releasing hormone, CRH, TAC1, prodynorphin, PDYN and tachykinin, TAC1) also promoted sleep. Many of these neurons project to the tuberomammillary nucleus (TMN), which releases the neuromodulator histamine. In contrast, glutaminergic POA neurons projecting to TMN induced wakefulness (Kroeger et al., 2018). Over the years, many sleep promoting neuron types and "sleep circuits" have been described (Weber and Dan, 2016). Yet, it is unlikely that sleep induction is the sole physiological function of these circuits.

Note 7. Blood pressure and heart rate decrease during nonREM sleep. This brain-body relationship between cardiovascular changes and sleep is reciprocal. Chemogenetic or optogenetic activation of barosensitive neurons in the nucleus of the solitary tract (NST) promotes non-REM sleep in addition to decreasing the blood pressure and heart rate (Koch 1932; Bridgers et al.,1985; Silvani and Dampney, 2013; Benarroch, 2018; Silvani et al., 2015; Yao et al., 2022). Cholinergic neurons in the nucleus ambiguus – one of the targets of the NST for cardiac baroreflex – also increased non-REM sleep. Furthemore, stimulation of the carotid baroreceptors in the body can be used to induce or facilitate sleep (Mazzella et al., 1957; Padel and Dell, 1965; Cooper and Hainsworth, 2009). In humans, the sleeping (supine) posture causes stronger activation of the baroreflex pathway (Cole 1989; no wonder we have a hard time sleeping in sitting, upright position on aircrafts).

Note 8. It should be noted that the POA is also regarded as the most important thermoregulatory "center" in the brain and also plays a role in energy conservation (Harding et al., 2018). These neurons receive indirect inputs from surface thermoreceptors in the skin, spinal cord, and deep structures in the abdominal cavity, and subgroups of POA neurons (expressing TRPM2 channels; Song et al., 2016) have the intrinsic ability to sense changes in local brain temperature. POA warming reduces heat production and increases heat-loss responses by vasodilation, sweating, increased respiration, inhibition of UCP-1 in brown adipose tissue. Moreover, it induces ambulation, search for cooler environment and, social crowding and nest building. Conversely, cooling of the same area increases heat production and reduces heat loss by inducing vasoconstriction, shivering, activation of UCP-1, increasing metabolism, and induces both locomotion and food intake (Siemens and Kamm, 2018). Thus, several functions are attributed to POA, although they may be interconnected. This interconnectedness feature is generalizable to other hypothalamic nuclei (and likely to thalamus and other brain regions). "Dedicatedness" is thus largely a reference to the investigating conditions rather than a reflection of true physiological function. This experimenter-centric classification is explicitly demonstrated by the discovery of the orexin/hypocretin-expressing neurons. One group of researchers, specialized in metabolism and feeding behavior, found a group of neurons in the lateral hypothalamus whose activity was correlated with appetite and eating and called these neurons orexin neurons (*orexis* means appetite; orexin-A and orexin-B subtypes; Sakurai et al., 1998). Another group of investigators independently identified these neurons as the pro-hormone pre-prohypocretin, and its peptide products hypocretin-1 (Hcrt-1) and hypocretin-2 (Hcrt-2; de Lecea et al., 1998; Peyron et al., 1998). Since these neurons project to many areas traditionally involved in arousal, vigilance and attention (cholinergic neurons of the basal forebrain, locus coeruleus,

dopaminergic neurons, reticular formation, amygdala, thalamus and basal ganglia; van den Pool et al., 1998) and because these peptides were abnormal in patients with narcolepsy (Nishino et al., 2000; van den Pol 2000), they concluded that the orexin system is a key to wake-sleep regulation. Intensive research over the past three decades expanded these initially "selective" and "specific" roles of orexin/hypocretin to numerous other functions, including regulation of water balance and energy, gastrointestinal and cardiovascular system control, neuroendocrine control, modulation of sensation and pain, gastric acid secretion and increases gut motility, sexual arousal, learning and Parkinson disease. The widespread inputs from numerous structures to the hypothalamus can account for the observation why stimulation of the same group of hypothalamic neurons can be linked to drinking, eating, knowing, object following, stalking attack or defensive behavior, likely determined by the circumstances (Valenstein et al., 1968). Brain circuits are rarely dedicated to a particular "function". Instead, their activity can induce a variety of related and overlapping behaviors, depending on the immediate affordances of the environment. Ideally, future experiments should include many external, body and brain measurements to compare and contrast the deduced correlations and results of perturbations. A complementary approach to this acknowledged high complexity of holistic monitoring is to study neuronal circuits in "simpler" organism in which the descending control on fundamental circuits and neurons is less complex (Marder et al., 2022).

Note 9. Brain temperature also has a profound effect on neurons and their physiological interactions. Most epileptic episodes occur prior to wakening, when brain temperature increases most steeply. Diurnal changes in of brain temperature is related to the variation of cluster headache, and abnormal rhythmicity of brain temperature predicts neurodegeneration and dysmenorrhea (Rzechorzek et al., 2022).

Note 10. Additionally, the hippocampus is privileged in its uptake of several peripherally originating messengers via the blood stream (Lathe et al., 2020). Whether via blood-brain or blood-CSF-brain transport, the list of compounds capable of reaching hippocampal circuits continues to grow and includes corticosterone (McEwen et al., 1997), leptin (personal communication P. Kalugin and M. Andermann), IGF-1 (Carro et al., 2000), IGF-2 (Chen et al., 2011; Stern et al., 2014), Ghrelin (Diano et al., 2006), insulin (Gray et al., 2017b; McNay et al., 2010; Park et al., 2000), progesterone (Guerra-Araiza et al., 2003, 2002; Kato et al., 1994), various cytokines (Banks et al., 1995; Gutierrez et al., 1993), and many peptides (Banks and Kastin, 1985).

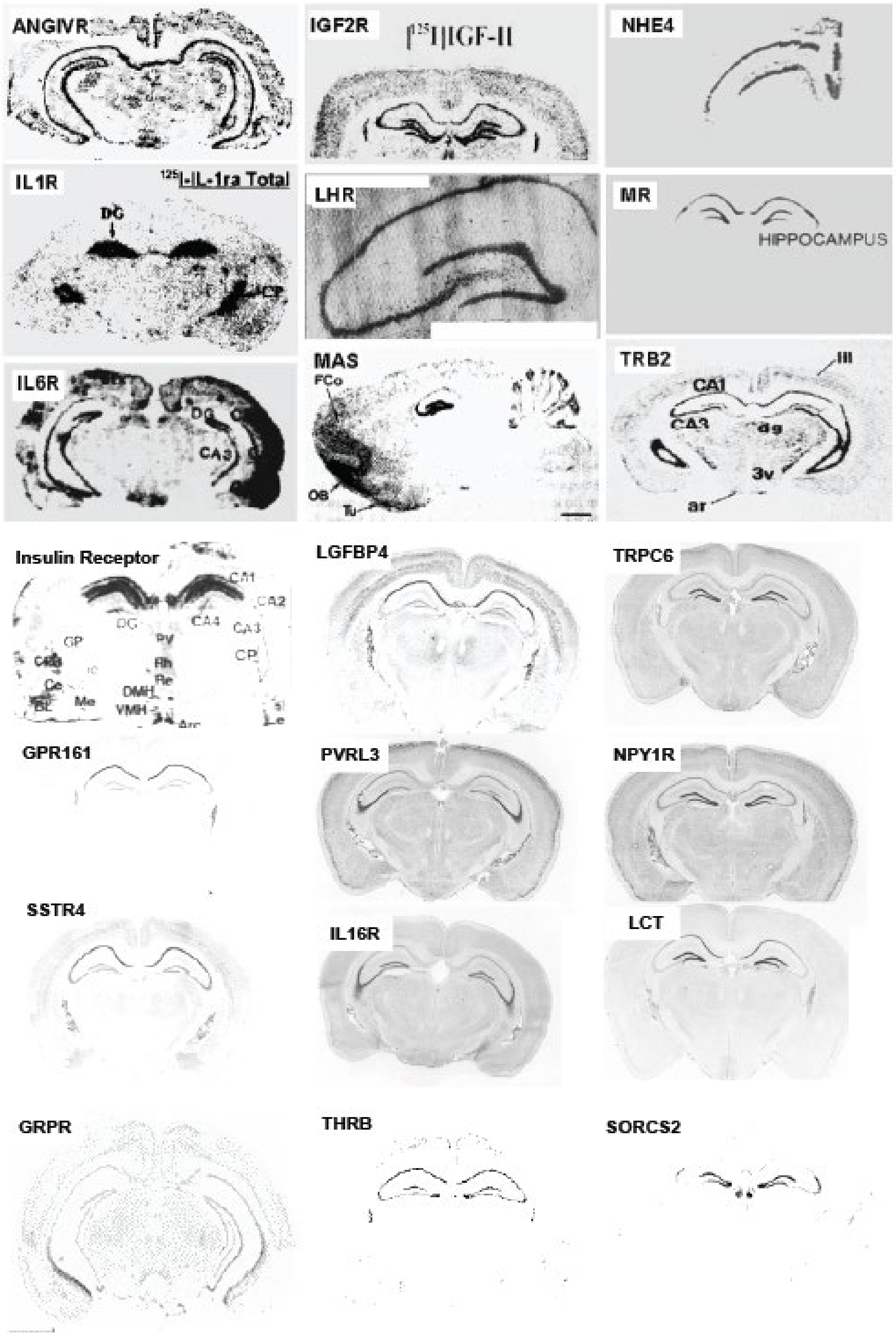

Supplementary Fig. 3. Inspired by (Lathe 2001), the hippocampus expresses a large number of receptors for 'non-canonical' signaling molecules. Reproduced from Lathte 2001. A modern-day version of this figure is the gene distribution atlas by Lein et al., (2007).

Alhadeff AL. Monitoring In Vivo Neural Activity to Understand Gut-Brain Signaling. Endocrinology. 2021 May 1;162(5):bqab029.

Andermann ML, Lowell BB. Toward a Wiring Diagram Understanding of Appetite Control. Neuron. 2017 Aug 16;95(4):757-778.

Baker FC, Driver HS, Rogers GG, Paiker J, Mitchell D. High nocturnal body temperatures and disturbed sleep in women with primary dysmenorrhea. Am J Physiol. 1999;277:E1013–E1021.

Banks, W.A., Kastin, A.J., 1985. Permeability of the blood-brain barrier to neuropeptides: the case for penetration. Psychoneuroendocrinology 10, 385–399.

Banks, W.A., Kastin, A.J., Broadwell, R.D., 1995. Passage of cytokines across the blood-brain barrier. Neuroimmunomodulation 2, 241–248. https://doi.org/10.1159/000097202

Benarroch, E.E. (2018). Brainstem integration of arousal, sleep, cardiovascular, and respiratory control. Neurology 91, 958–966.

Benedetti F. Placebo Effects: Understanding the mechanisms in health and disease. Oxford University Press. 2008.

Besedovsky, H.O., del Rey, A., Klusman, I., Furukawa, H., Monge Arditi, G., Kabiersch, A., 1991. Cytokines as modulators of the hypothalamus-pituitary-adrenal axis. J. Steroid Biochem. Mol. Biol. 40, 613–618.

Bierwolf, C., Struve, K., Marshall, L., Born, J., Fehm, H.L., 1997. Slow wave sleep drives inhibition of pituitary-adrenal secretion in humans. J. Neuroendocrinol. 9, 479–484.

Born J, Wagner U. Memory consolidation during sleep: role of cortisol feedback. Ann N Y Acad Sci. 2004. 1032:198-201.

Born, J., Fehm, H.L., 1998. Hypothalamus-pituitary-adrenal activity during human sleep: a coordinating role for the limbic hippocampal system. Exp. Clin. Endocrinol. Diabetes Off. J. Ger. Soc. Endocrinol. Ger. Diabetes Assoc. 106, 153–163.

Bridgers, S.L., S. S. Spencer, D. D. Spencer, C. T. Sasaki, A cerebral effect of carotid sinus stimulation. Observation during intraoperative electroencephalographic monitoring. Arch Neurol 42, 574-577 (1985).

Carro, E., Nuñez, A., Busiguina, S., Torres-Aleman, I., 2000. Circulating insulin-like growth factor I mediates effects of exercise on the brain. J. Neurosci. Off. J. Soc. Neurosci. 20, 2926–2933.

Chen, D.Y., Stern, S.A., Garcia-Osta, A., Saunier-Rebori, B., Pollonini, G., Bambah-Mukku, D., Blitzer, R.D., Alberini, C.M., 2011. A critical role for IGF-II in memory consolidation and enhancement. Nature 469, 491–497.

Chung, S., Weber, F., Zhong, P., Tan, C. L., Nguyen, T., Beier, K. T., et al. (2017). Identification of preoptic sleep neurons using retrograde labeling and gene profiling. Nature 545, 477–481. Cooper VL, Hainsworth R. Carotid baroreflex testing using the neck collar device. Clin Auton Res 19, 102-112 (2009).

Cole, R.J. (1989). Postural baroreflex stimuli may affect EEG arousal and sleep in humans. J. Appl. Physiol. (1985) 67, 2369–2375.

Czeisler, C. A., Weitzman, E. D., Moore-Ede, M. C., Zimmerman, J. C. & Knauer, R. Science 210, 1264–1267 (1980).

D'Amato A, Di Cesare Mannelli L, Lucarini E, Man AL, Le Gall G, Branca JJV, Ghelardini C, Amedei A, Bertelli E, Regoli M, Pacini A, Luciani G, Gallina P, Altera A, Narbad A, Gulisano M, Hoyles L, Vauzour D, Nicoletti C. Faecal microbiota transplant from aged donor mice affects spatial learning and memory via modulating hippocampal synaptic plasticity- and neurotransmission-related proteins in young recipients. Microbiome. 2020. 8(1):140.

Dana, R. C., & Martinez, J. L., Jr. (1984). Effect of adrenalectomy on the circadian rhythm of LTP. Brain Research, 308, 392–395.

de Lecea L, Kilduff TS, Peyron C, Gao X, Foye PE, Danielson PE, Fukuhara C, Battenberg EL, Gautvik VT, Bartlett FS 2nd, Frankel WN, van den Pol AN, Bloom FE, Gautvik KM, Sutcliffe JG. The hypocretins: hypothalamus-specific peptides with neuroexcitatory activity. Proc Natl Acad Sci USA 1998;95: 322-7

Deinzer, R., Kirschbaum, C., Gresele, C. & Hellhammer, H. Physiol. Behav. 61, 507–511 (1997).

Diano, S., Farr, S.A., Benoit, S.C., McNay, E.C., da Silva, I., Horvath, B., Gaskin, F.S., Nonaka, N., Jaeger, L.B., Banks, W.A., Morley, J.E., Pinto, S., Sherwin, R.S., Xu, L., Yamada, K.A., Sleeman, M.W., Tschöp, M.H., Horvath, T.L., 2006. Ghrelin controls hippocampal spine synapse density and memory performance. Nat. Neurosci. 9, 381–388.

Ebrahim IO, Howard RS, Kopelman MD, Sharief MK, Williams AJ. The hypocretin/orexin system. J R Soc Med. 2002. 95(5):227-30.

Endroczi, E., Lissak, K., Bohus, B., Kovacs, S., 1959. Inhibitory influnce of archicortical structures on pituitary-adrenal function. Physiologia XVI, 2–20.

Fisher, S., Cui, N., McKillop, L. et al. Stereotypic wheel running decreases cortical activity in mice. Nat Commun 7: 13138 (2016).

Freund TF, Buzsáki G. Interneurons of the hippocampus. Hippocampus. 1996;6(4):347-470.

Gautvik KM, de Lecea L, Gautvik VT, *et al*. Overview of the most prevalent hypothalamus-specific mRNAs, as identified by directional tag PCR subtraction. Proc Natl Acad Sci USA 1996;93: 8733-8

Gray, S.M., Aylor, K.W., Barrett, E.J., 2017b. Unravelling the regulation of insulin transport across the brain endothelial cell. Diabetologia 60, 1512–1521.

Gray, S.M., Meijer, R.I., Barrett, E.J., 2014. Insulin regulates brain function, but how does it get there? Diabetes 63, 3992–3997.

Guerra-Araiza, C., Coyoy-Salgado, A., Camacho-Arroyo, I., 2002. Sex differences in the regulation of progesterone receptor isoforms expression in the rat brain. Brain Res. Bull. 59, 105–109.

Guerra-Araiza, C., Villamar-Cruz, O., González-Arenas, A., Chavira, R., Camacho-Arroyo, I., 2003. Changes in Progesterone Receptor Isoforms Content in the Rat Brain During the Oestrous Cycle and After Oestradiol and Progesterone Treatments. J. Neuroendocrinol. 15, 984–990.

Gutierrez, E.G., Banks, W.A., Kastin, A.J., 1993. Murine tumor necrosis factor alpha is transported from blood to brain in the mouse. J. Neuroimmunol. 47, 169–176.

Harding EC, Yu X, Miao A, Andrews N, Ma Y, Ye Z, Lignos L, Miracca G, Ba W, Yustos R, Vyssotski AL, Wisden W, Franks NP. A Neuronal Hub Binding Sleep Initiation and Body Cooling in Response to a Warm External Stimulus. Curr Biol. 2018. 28(14):2263-2273.

Hastings M, O'Neill JS, Maywood ES. Circadian clocks: regulators of endocrine and metabolic rhythms. J Endocrinol. 2007;195:187–198.

Hobson, J. A. & Stickgold, R. in The Cognitive Neurosciences (ed. Gazzaniga, M.) 1373–1389 (MIT Press, Cambridge, MA, 1995).

Ibberson, M., Riederer, B.M., Uldry, M., Guhl, B., Roth, J., Thorens, B., 2002.

Immunolocalization of GLUTX1 in the testis and to specific brain areas and vasopressin-containing neurons. Endocrinology 143, 276–284.

Kaelberer MM, Buchanan KL, Klein ME, Barth BB, Montoya MM, Shen X, Bohórquez DV. A gut-brain neural circuit for nutrient sensory transduction. Science. 2018. 361(6408):eaat5236.

Karoly PJ, Goldenholz DM, Freestone DR, et al. Circadian and circaseptan rhythms in human epilepsy: a retrospective cohort study. Lancet Neurol. 2018;17:977–985. 2286–2294.

Kilduff TS, Peyron C. The hypocretin/orexin ligand-receptor system: implications for sleep and sleep disorders. TINS 2000;23: 359-64.

Koch, E. Die Irradiation der Pressoreceptorischen Kreislaufreflexe. Klinische Wochenschrift 11, 225-227 (1932).

Kroeger, D., Absi, G., Gagliardi, C., Bandaru, S. S.,Madara, J. C., Ferrari, L. L., et al. (2018). Galanin neurons in the ventrolateral preoptic area promote sleep and heat loss in mice. Nat. Commun. 9:4129. doi: 10.1038/s41467-018-06590-7

Lathe, R., Singadia, S., Jordan, C., Riedel, G., 2020. The interoceptive hippocampus: Mouse brain endocrine receptor expression highlights a dentate gyrus (DG)–cornu ammonis (CA) challenge–sufficiency axis. PLOS ONE 15, e0227575.

LeDoux JE. Emotion: clues from the brain. Annu Rev Psychol. 1995;46:209-35.

Leproult R, Copinschi G, Buxton O, Van Cauter E. Sleep loss results in an elevation of cortisol levels the next evening. Sleep. 1997;**20**(10):865–870.

Liu D, Dan Y. A Motor Theory of Sleep-Wake Control: Arousal-Action Circuit. Annu Rev Neurosci. 2019 Jul 8;42:27-46.

Lodi R, Pierangeli G, Tonon C, et al. Study of hypothalamic metabolism in cluster headache by proton MR spectroscopy. Neurology. 2006;66:1264–1266.

Lyamin OI, Mukhametov LM, Siegel JM. Relationship between sleep and eye state in Cetaceans and Pinnipeds. Arch Ital Biol. 2004 Jul;142(4):557-68.

MacDonald, G.; Leary, M.R. (2005). "Why does social exclusion hurt? The relationship between social and physical pain". Psychological Bulletin. **131** (2): 202–23.

Marder E, Haddad SA, Goeritz ML, Rosenbaum P, Kispersky T. How can motor systems retain performance over a wide temperature range? Lessons from the crustacean stomatogastric nervous system. J Comp Physiol A Neuroethol Sens Neural Behav Physiol. 2015 Sep;201(9):851-6.

Marder E, Kedia S, Morozova EO. New insights from small rhythmic circuits. Curr Opin Neurobiol. 2022 Oct;76:102610.

Mason JW, Hartley LH, Kotchen TA, Mougey EH, Ricketts PT, Jones LG. Plasma cortisol and norepinephrine responses in anticipation of muscular exercise. Psychosom Med. 1973. 35(5):406-14.

May A, Bahra A, Büchel C, Frackowiak RS, Goadsby PJ. Hypothalamic activation in cluster headache attacks. Lancet. 1998;352:275–278.

Mazzella, H., Garcia Mullin, R., and Garcia Austt, E. (1957). Effect of carotid sinus stimulation on the EEG. Acta Neurol. Latinoam. 3, 361–364.

McEwen, B.S., Conrad, C.D., Kuroda, Y., Frankfurt, M., Magarinos, A.M., McKittrick, C., 1997. Prevention of stress-induced morphological and cognitive consequences. Eur. Neuropsychopharmacol. J. Eur. Coll. Neuropsychopharmacol. 7 Suppl 3, S323-328.

McGinty, D. J. & Sterman, M. B. Sleep suppression after basal forebrain lesions in the cat. Science **160**, 1253–1255 (1968)

McNay, E.C., Ong, C.T., McCrimmon, R.J., Cresswell, J., Bogan, J.S., Sherwin, R.S., 2010. Hippocampal memory processes are modulated by insulin and high-fat-induced insulin resistance. Neurobiol. Learn. Mem. 93, 546–553. https://doi.org/10.1016/j.nlm.2010.02.002

Moorcroft WH, Kayser KH, Griggs AJ. Subjective and objective confirmation of the ability to self-awaken at a self-predetermined time without using external means. Sleep. 1997. 20(1):40-5

Moser E, Mathiesen I, Andersen P. Association between brain temperature and dentate field potentials in exploring and swimming rats. Science. 1993;259:1324–1326.

Nauta, W. J. Hypothalamic regulation of sleep in rats; an experimental study. J. Neurophysiol. **9**, 285–316 (1946).

Nishino S, Ripley B, Overeem S, Lammers GJ, Mignot E. Hypocretin (orexin) deficiency in human narcolepsy. Lancet. 2000 Jan 1;355(9197):39-40.

Padel, Y., and Dell, P. (1965). Effets bulbaires et réticulaires des stimulations endormantes du tronc vago-aortique. J. Physiol. Paris 57, 269–270.

Park, C.R., Seeley, R.J., Craft, S., Woods, S.C., 2000. Intracerebroventricular insulin enhances memory in a passive-avoidance task. Physiol. Behav. 68, 509–514.

Parra P, Gulyás AI, Miles R. How many subtypes of inhibitory cells in the hippocampus? Neuron. 1998 May;20(5):983-93

Petersen PC, Vöröslakos M, Buzsáki G. Brain temperature affects quantitative features of hippocampal sharp wave ripples. J Neurophysiol. 2022 May 1;127(5):1417-1425.

Petersen, P.C., Siegle, J.H., Steinmetz, N.A., Mahallati, S., Buzsáki, G., 2021. CellExplorer: A framework for visualizing and characterizing single neurons. Neuron 109, 3594-3608.e2.

Petreanu, L., Gutnisky, D.A., Huber, D., Xu, N., O'Connor, D.H., Tian, L., Looger, L., Svoboda, K., 2012. Activity in motor-sensory projections reveals distributed coding in somatosensation. Nature 489, 299–303.

Peyron C, Tighe DK, van den Pol AN, de Lecea L, Heller HC, Sutcliffe JG, Kilduff TS. Neurons containing hypocretin (orexin) project to multiple neuronal systems. J Neurosci. 1998. 18(23):9996-10015.

Rango M, Bonifati C, Bresolin N. Post-activation brain warming: a thermometry study. PLoS One. 2015;10:e0127314.

Rossi S, Zanier ER, Mauri I, Columbo A, Stocchetti N. Brain temperature, body core temperature, and intracranial pressure in acute cerebral damage. J Neurol Neurosurg Psychiatry. 2001;71: 448–454.

Rzechorzek NM, Thrippleton MJ, Chappell FM, Mair G, Ercole A, Cabeleira M; CENTER-TBI High Resolution ICU (HR ICU) Sub-Study Participants and Investigators, Rhodes J, Marshall I,

O'Neill JS. A daily temperature rhythm in the human brain predicts survival after brain injury. Brain. 2022 Jun 13:awab466

Sakurai T, Amemiya A, Ishii M, *et al*. Orexins and orexin receptors: a family of hypothalamic neuropeptides and G-protein coupled receptors that regulate feeding behaviour. Cell 1998;92: 573-5

Siemens J, Kamm GB. Cellular populations and thermosensing mechanisms of the hypothalamic thermoregulatory center. Pflugers Arch. 2018 May;470(5):809-822.

Silvani, A., Calandra-Buonaura, G., Benarroch, E.E., Dampney, R.A., and Cortelli, P. (2015). Bidirectional interactions between the baroreceptor reflex and arousal: an update. Sleep Med. 16, 210–216.

Silvani, A., and Dampney, R.A. (2013). Central control of cardiovascular function during sleep. Am. J. Physiol. Heart Circ. Physiol. 305, H1683–H1692.

Song K, Wang H, Kamm GB, Pohle J, Reis FC, Heppenstall P, Wende H, Siemens J. The TRPM2 channel is a hypothalamic heat sensor that limits fever and can drive hypothermia. Science. 2016 Sep 23;353(6306):1393-1398.

Späth-Schwalbe, E., Schöller, T., Kern, W., Fehm, H. L. & Born, J. J. Clin. Endocrinol. Metab. 75, 1431–1435 (1992).

Stern, S.A., Chen, D.Y., Alberini, C.M., 2014. The effect of insulin and insulin-like growth factors on hippocampus- and amygdala-dependent long-term memory formation. Learn. Mem. Cold Spring Harb. N 21, 556–563.

Suarez AN, Hsu TM, Liu CM, Noble EE, Cortella AM, Nakamoto EM, Hahn JD, de Lartigue G, Kanoski SE. Gut vagal sensory signaling regulates hippocampus function through multi-order pathways. Nat Commun. 2018. 9(1):2181.

Sutcliffe JG, de Lecea L. The hypocretins: excitatory neuromodulatory peptides for multiple homeostatic systems, including sleep and feeding. J Neurosci Res 2000. 62:161-8

Thannickal TC, Lai YY, Siegel JM. Hypocretin (orexin) cell loss in Parkinson's disease. Brain. 2007. 130:1586-95.

Trivedi P, Yu H, MacNeil DJ, Van der Ploeg LH, Guan XM. Distribution of orexin receptor mRNA in the rat brain. FEBS Lett 1998;438: 71-59. 9. Chicurel M. The sandman's secrets. Nature 2000;407: 554-6

Van Cauter, E. & Turek, F. W. in Endocrinology Vol. 3 (ed. DeGroot, L. J.) 2487–2548 (Saunders, Philadelphia, 1995).

van den Pol AN, Gao XB, Obrietan K, Kilduff TS, Belousov AB. Presynaptic and postsynaptic actions and modulation of neuroendocrine neurons by a new hypothalamic peptide, hypocretin/orexin. J Neurosci. 1998. 18(19):7962-71.

van den Pol AN. Narcolepsy: a neurodegenerative disease of the hypocretin system? Neuron. 2000 Sep;27(3):415-8.

Von Economo, C. Sleep as a problem of localization. J. Nerv. Ment. Dis. **71**, 249–259 (1930)

Vosko, A.M., Schroeder, A., Loh, D.H., Colwell, C.S., 2007. Vasoactive intestinal peptide and the mammalian circadian system. Gen. Comp. Endocrinol. 152, 165–175.

Yao Y, Barger Z, Saffari Doost M, Tso CF, Darmohray D, Silverman D, Liu D, Ma C, Cetin A, Yao S, Zeng H, Dan Y. Cardiovascular baroreflex circuit moonlights in sleep control. Neuron. 2022 Dec 7;110(23):3986-3999.

Closing the Loop

Note 1. There are several predictions that can be made for this glucose regulatory body-brain-body control loop. While it remains to be seen whether such a loop employs positive or negative feedback, homeostatic demands employ negative feedback loops far more often (Cannon 1963; Robertson 1991). Initial data suggests that the direct action of insulin on the hippocampus is to suppress network excitability (Jin et al. 2011; O'Malley et al. 2003; O'Malley & Harvey 2004; Palovcik et al. 1984; Zhao et al. 2019), whereas during SPW-R the hippocampus is in its most excitable state, suggesting this loop may enact negative feedback with SPW-R induced insulin secretion leading to the cessation of further SPW-Rs. The presence of such a control loop would also predict that by suppressing SPW-R activity—and thus disconnecting this form of negative feedback—a homeostatic counter-regulatory drive would be to increase the rate of SPW-Rs (Girardeau et al. 2014).

Cannon W. 1963. The Wisdom of The Body. W. W. Norton & Company

Girardeau G, Cei A, Zugaro M. Learning-induced plasticity regulates hippocampal sharp wave-ripple drive. J Neurosci. 2014 Apr 9;34(15):5176-83.

Jin Z, Jin Y, Kumar-Mendu S, Degerman E, Groop L, Birnir B. 2011. Insulin reduces neuronal excitability by turning on GABAA channels that generate tonic current. *PLoS ONE*. 6(1):1–8

Lathe R. 2001. Hormones and the hippocampus. *J Endocrinol*. 169(2):205–31

O'Malley D, Harvey J. 2004. Insulin activates native and recombinant large conductance Ca 2+-activated potassium channels via a mitogen-activated protein kinase-dependent process. *Molecular Pharmacology*. 65(6):1352–63

O'Malley D, Shanley LJ, Harvey J. 2003. Insulin inhibits rat hippocampal neurones via activation of ATP-sensitive K+ and large conductance Ca2+-activated K+ channels. *Neuropharmacology*. 44(7):855–63

Palovcik RA, Phillips MI, Kappy MS, Raizada MK. 1984. Insulin inhibits pyramidal neurons in hippocampal slices. *Brain Res*. 309(1):187–91

Robertson DS. 1991. Feedback theory and Darwinian evolution. *Journal of Theoretical Biology*. 152(4):469–84

Seto K, Saito H, Otsuka K, Kawakami M. 1983. Influence of electrical stimulation of the limbic structure on insulin level in rabbit's plasma. *Exp Clin Endocrinol*. 81(3):347–49

Taylor, K.S., Kucyi, A., Millar, P.J., Murai, H., Kimmerly, D.S., Morris, B.L., Bradley, T.D., Floras, J.S., 2016. Association between resting-state brain functional connectivity and muscle sympathetic burst incidence. J. Neurophysiol. 115, 662–673.

Van Cauter, E., Kerkhofs, M., Caufriez, A., Van Onderbergen, A., Thorner, M.O., Copinschi, G., 1992. A quantitative estimation of growth hormone secretion in normal man: reproducibility and relation to sleep and time of day. J. Clin. Endocrinol. Metab. 74, 1441–1450.

Zhao F, Siu JJ, Huang W, Askwith C, Cao L. 2019. Insulin Modulates Excitatory Synaptic Transmission and Synaptic Plasticity in the Mouse Hippocampus. *Neuroscience*. 411:237–54

EXAPTATION OF MEMORY FROM NAVIGATION

Lever, C., Burton, S., Jeewajee, A., O'Keefe, J., Burgess, N., 2009. Boundary vector cells in the subiculum of the hippocampal formation. J. Neurosci. Off. J. Soc. Neurosci. 29, 9771–9777.

McClain, K., Tingley, D., Heeger, D.J., Buzsáki, G., 2019. Position–theta-phase model of hippocampal place cell activity applied to quantification of running speed modulation of firing rate. Proc. Natl. Acad. Sci. 116, 27035–27042.

Sargolini, F., Fyhn, M., Hafting, T., McNaughton, B.L., Witter, M.P., Moser, M.-B., Moser, E.I., 2006. Conjunctive representation of position, direction, and velocity in entorhinal cortex. Science 312, 758–762.

Scavuzzo, C.J., Newman, L.A., Gold, P.E., Korol, D.L., 2021. Extracellular levels of glucose in the hippocampus and striatum during maze training for food or water reward in male rats. Behav. Brain Res. 411, 113385.

Solstad, T., Boccara, C.N., Kropff, E., Moser, M.-B., Moser, E.I., 2008. Representation of geometric borders in the entorhinal cortex. Science 322, 1865–1868.

Tulving, E., 2005. Episodic Memory and Autonoesis: Uniquely Human?. In: The Missing Link in Cognition: Origins of Self-Reflective Consciousness. Oxford University Press, New York, NY, US, pp. 3–56.

CONCLUSIONS AND OUTLOOK

Note 1. A recommended practical step forward is to distinguish between paradigm-independent and paradigm-dependent changes in brain correlates in experimental studies. Properties of neuronal activity, e.g., spikes, can be described at multiple levels of complexity. The first level is a description of their biophysical characteristics, molecular identity, transmitter type, relationship to spikes of other neurons (e.g., synchrony) and local field potentials. This level may also include other metrics related to firing patterns, such as interspike interval statistics and autocorrelograms. Next, spiking activity can be related to brain states (e.g., non-rapid-eye-movement [non-REM], REM, awake states and their transitions or a continuous variable quantifying these states) and overt behavioral correlates. The latter can include spontaneous motor patterns, movement pattern changes, locomotion speed, head turns, whisker movements, respiration, heart rate, body temperature, pupil diameter, and other autonomic parameters, and, possibly, pH, ion concentration, osmolarity and humoral factors in the body. These first order descriptions provide generic features of neuronal activity common to all experimental paradigms in the same species and, therefore, are communicable across different experiments and laboratories, leading to joint databases and standardized metrics across different laboratories (Petersen et al., 2021). Ideally, only after these paradigm-independent features are established, should we turn to the relationship between the observed brain parameters and experiment-unique manipulations. Because these latter correlations are paradigm-specific and differ across laboratories, the first-order analyses can safeguard against inappropriately attributing observed neuronal patterns to high-level phenomena, such as learning, perception, or decision making, each of which are often linked to overt movement and autonomic changes.

Note 2. *The depth and breadth of monitoring body-brain interactions.* When studying brains, or brains within bodies, it is constructive to conceptualize them as dynamical systems where each component is a node within an interconnected graph. These ‘components’ are largely defined by

one's level of interest, and are typically depicted as a neuron, circuit or brain region. In each of these cases, however, the connection between nodes almost universally is thought of as synaptic. Additionally, the experiments we design to study these systems typically collect two or a few modalities (e.g., calcium imaging and stimulus presentation, or spiking activity and behavioral tracking).

This conceptualization and style of experiment has driven a particular type of tool development in past decades which we refer to as ***depth*** (Machado et al., 2022). That is, our ability to measure calcium activity or spiking activity from populations of neurons grows on a roughly exponential scale, doubling every ~6 years (Steinmetz et al., 2018; Stevenson, 2020). Ignoring the constraints of tissue damage, this would mean that simultaneous spiking activity from ~1 million neurons will be recorded in approximately 50 years. While the utility of such datasets have been previously discussed (Hasselmo, 2015), such experiments are inherently limited by the ***breadth*** of observations available. Is this neuron firing because of the stimulus being presented? Or due to some other unobserved physiological parameter such as respiration, heart rate, temperature, metabolic state, neuromodulatory tone or intrinsic dynamics?

In contrast to the ever-increasing depth of observability, the breadth of observations typically made in a neuroscientific experiment has not progressed in nearly 100 years and remains on merely a handful of unique types of signals (Adrian and Zotterman, 1926; Allen et al., 2019; Inagaki et al., 2022; Stringer et al., 2019).

While other forms of biosensing—besides calcium and extracellular action potentials—have a rich history (Bergveld, 1970; Clark Jr. and Lyons, 1962; Cremer, 1906; Hughes, 1922; Robinson et al., 2003), tool development in this space has seen a renewed interest with the development of biosensors for neurotransmitters (Marvin et al., 2018), neuromodulators (Bulumulla and Beyene, 2022; Wang et al., 2022; Wu et al., 2022), and various other peptides (Wang et al., 2022). However, it is critical to highlight that each of these tools affords the addition of a single modality, and rarely have been combined to simultaneously measure multiple signals in a single experiment. We propose that a tool development program which emphasizes ***observational breadth*** by engineering the development of multiplexed biosensors will help to unlock new understanding the relevance of brain activity in appropriate ecological settings. Such tools will allow for a most clear picture of how many different biological signals may be integrated and lead to a brain mechanism-based classification of cognitive behaviors (Fig. 5).

Berry RJ, Bronson FH. Life history and bioeconomy of the house mouse. Biol Rev Camb Philos Soc. 1992 Nov;67(4):519-50.

Crowcroft P. Mice All Over. Chicago Zoological Society. 1973

Giancarlo Fortino, Hassan, Wenfeng, Yin Zhang, Luca Benini (editors) Special Issue "Advances in Body Sensor Networks: Sensors, Systems, and Applications". Sensors 2017, vol. *17*.

Keramati, M., Gutkin, B., 2014. Homeostatic reinforcement learning for integrating reward collection and physiological stability. eLife 3, e04811.

Lin R, Kim HJ, Achavananthadith S, Kurt SA, Tan SCC, Yao H, Tee BCK, Lee JKW, Ho JS. Wireless battery-free body sensor networks using near-field-enabled clothing. Nat Commun. 2020 Jan 23;11(1):444.

Marshall, J.D., Aldarondo, D.E., Dunn, T.W., Wang, W.L., Berman, G.J., Ölveczky, B.P., 2021. Continuous Whole-Body 3D Kinematic Recordings across the Rodent Behavioral Repertoire. Neuron 109, 420-437.

Miller, C.T., Gire, D., Hoke, K., Huk, A.C., Kelley, D., Leopold, D.A., Smear, M.C., Theunissen, F., Yartsev, M., Niell, C.M., 2022. Natural behavior is the language of the brain. Curr. Biol. 32, R482–R493.

Parker PRL, Brown MA, Smear MC, Niell CM. Movement-Related Signals in Sensory Areas: Roles in Natural Behavior. Trends Neurosci. 2020 Aug;43(8):581-595.

Yuce MR. Recent Wireless Body Sensors: Design and Implementation. IEEE MTT-S International Microwave Workshop Series on RF and Wireless Technologies for Biomedical and Healthcare Applications (IMWS-BIO). 2013. DOI: 10.1109/IMWS-BIO.2013.6756254
